# Mechanisms for the circulation of influenza A(H3N2) in China: A spatiotemporal modelling study

**DOI:** 10.1371/journal.ppat.1011046

**Published:** 2022-12-16

**Authors:** Bing Zhang, Weijuan Huang, Sen Pei, Jinfeng Zeng, Wei Shen, Daoze Wang, Gang Wang, Tao Chen, Lei Yang, Peiwen Cheng, Dayan Wang, Yuelong Shu, Xiangjun Du

**Affiliations:** 1 School of Public Health (Shenzhen), Sun Yat-sen University, Guangzhou, People’s Republic of China; 2 School of Public Health (Shenzhen), Shenzhen Campus of Sun Yat-sen University, Shenzhen, People’s Republic of China; 3 Guangzhou First People’s Hospital, School of Medicine, South China University of Technology, Guangzhou, People’s Republic of China; 4 National Institute for Viral Disease Control and Prevention, Collaboration Innovation Center for Diagnosis and Treatment of Infectious Diseases, Chinese Center for Disease Control and Prevention, Beijing, People’s Republic of China; 5 Department of Environmental Health Sciences, Mailman School of Public Health, Columbia University, New York, United States of America; 6 Department of Rheumatology and Immunology, Drum Tower Clinic Medical College of Nanjing Medical University, Nanjing, People’s Republic of China; 7 Key Laboratory of Tropical Disease Control, Ministry of Education, Sun Yat-sen University, Guangzhou, People’s Republic of China; 8 Institute of Pathogen Biology of Chinese Academy of Medical Science (CAMS)/ Peking Union Medical College (PUMC), Beijing, People’s Republic of China; National Institutes of Health, UNITED STATES

## Abstract

Circulation of seasonal influenza is the product of complex interplay among multiple drivers, yet characterizing the underlying mechanism remains challenging. Leveraging the diverse seasonality of A(H3N2) virus and abundant climatic space across regions in China, we quantitatively investigated the relative importance of population susceptibility, climatic factors, and antigenic change on the dynamics of influenza A(H3N2) through an integrative modelling framework. Specifically, an absolute humidity driven multiscale transmission model was constructed for the 2013/2014, 2014/2015 and 2016/2017 influenza seasons that were dominated by influenza A(H3N2). We revealed the variable impact of absolute humidity on influenza transmission and differences in the occurring timing and magnitude of antigenic change for those three seasons. Overall, the initial population susceptibility, climatic factors, and antigenic change explained nearly 55% of variations in the dynamics of influenza A(H3N2). Specifically, the additional variation explained by the initial population susceptibility, climatic factors, and antigenic change were at 33%, 26%, and 48%, respectively. The vaccination program alone failed to fully eliminate the summer epidemics of influenza A(H3N2) and non-pharmacological interventions were needed to suppress the summer circulation. The quantitative understanding of the interplay among driving factors on the circulation of influenza A(H3N2) highlights the importance of simultaneous monitoring of fluctuations for related factors, which is crucial for precise and targeted prevention and control of seasonal influenza.

## Author summary

Seasonal influenza, especially for A(H3N2), has a considerable impact on public health around the world. Until now, quantitatively characterizing the underlying mechanism for the circulation of influenza A(H3N2) remains challenging, especially for countries with diverse seasonal patterns. China is a climatologically diverse country, where the seasonal pattern of influenza A(H3N2) varies across regions. Using multi-source data, the mechanism for the heterogenic transmission patterns of influenza A(H3N2) in China was revealed through an integrative modelling framework. The contributions of population susceptibility, climate factors and antigenic change to the dynamics of influenza A(H3N2) were also quantified. Finally, the effects of interventions on the summer epidemics were tested to help for optimizing the routine influenza immunization campaigns in China. The quantitative understanding of the interplay among driving factors on the circulation of influenza A(H3N2) highlights the importance of simultaneous monitoring of fluctuations for related factors, which is crucial for precise and targeted prevention and control of seasonal influenza.

## Introduction

Seasonal influenza is a major threat to public health, which infects 5–15% population and causes around 0.5 million deaths worldwide every year [1]. Influenza A(H3N2), as one subtype of the seasonal influenza virus, is the most recurring one causing repetitive epidemics [2]. Since its emergence in 1968, the influenza A(H3N2) virus has been continually evolving both genetically and antigenically, resulting in a considerable burden on human health [3]. Despite its global prevalence, a quantitative understanding of the mechanism underlying the circulation of influenza A(H3N2) virus remains incomplete, especially for countries (e.g., China) with diverse seasonal patterns [4].

A large number of studies demonstrated that the repetitive influenza A(H3N2) epidemics are determined to a large degree by the antigenic drift or shift of the surface glycoproteins (HA: hemagglutinin, NA: neuraminidase) [5]. On the other hand, due to the waning immunity, without new antigenic variants, the old influenza strains are capable of re-invading, leading to recurrent dynamics [6,7]. Since multiple infections of seasonal influenza virus are possible during the lifetime and antigenic variants continue to emerge, the effect of population immunity on influenza dynamics has been proven challenging to be characterized [3].

Climatic factors, especially absolute humidity (AH) [8], also have been considered as a critical driver in determining the transmission pattern of seasonal influenza [9]. The seasonal pattern of influenza epidemics varies globally, with the majority of infections occurring in the winter-spring months in temperate regions and during the rainy season in tropical regions [10]. This regional heterogeneity in influenza seasonality raises the question of which and how ecological factors are involved in the dynamics of seasonal influenza. Considering the nonlinear effects of climatic factors on influenza transmission [11], conclusions derived from studies based on a single location where a narrow climatic space exists could mislead the understanding of climatic dependency for seasonal influenza virus. Thus, more works concentrating on multi-region studies with abundant climatic spaces, are warranted.

China is a climatologically diverse country, where the seasonal pattern of influenza A(H3N2) varies across regions [4]. Previous studies have identified potential factors involved in the circulating of influenza, such as environmental forcing, holiday effect, population immunity and viral mutations [4,6,12]. However, these studies typically focused on just one or a subset of factors. As a result, it is still not clear about the relative importance of these driving factors on the dynamics of influenza A(H3N2), when multiple driving factors are considered simultaneously. To address these issues, utilizing the high-resolution surveillance data of seasonal influenza in China, we try to reveal the major drivers behind the dynamics of influenza A(H3N2) and quantify their relative contributions. Considering the heterogeneous effect of antigenic change on influenza transmission in different epidemic seasons, an integrative mathematical modelling framework across regions for several individual seasons was utilized here, instead of fitting a hierarchical model to pool data from a single region across seasons [6,13]. This special setup allows for better disentangling the relative roles of different drivers in influenza transmission.

## Results

### Circulation variation of influenza A(H3N2) across regions

**Fig 1:**
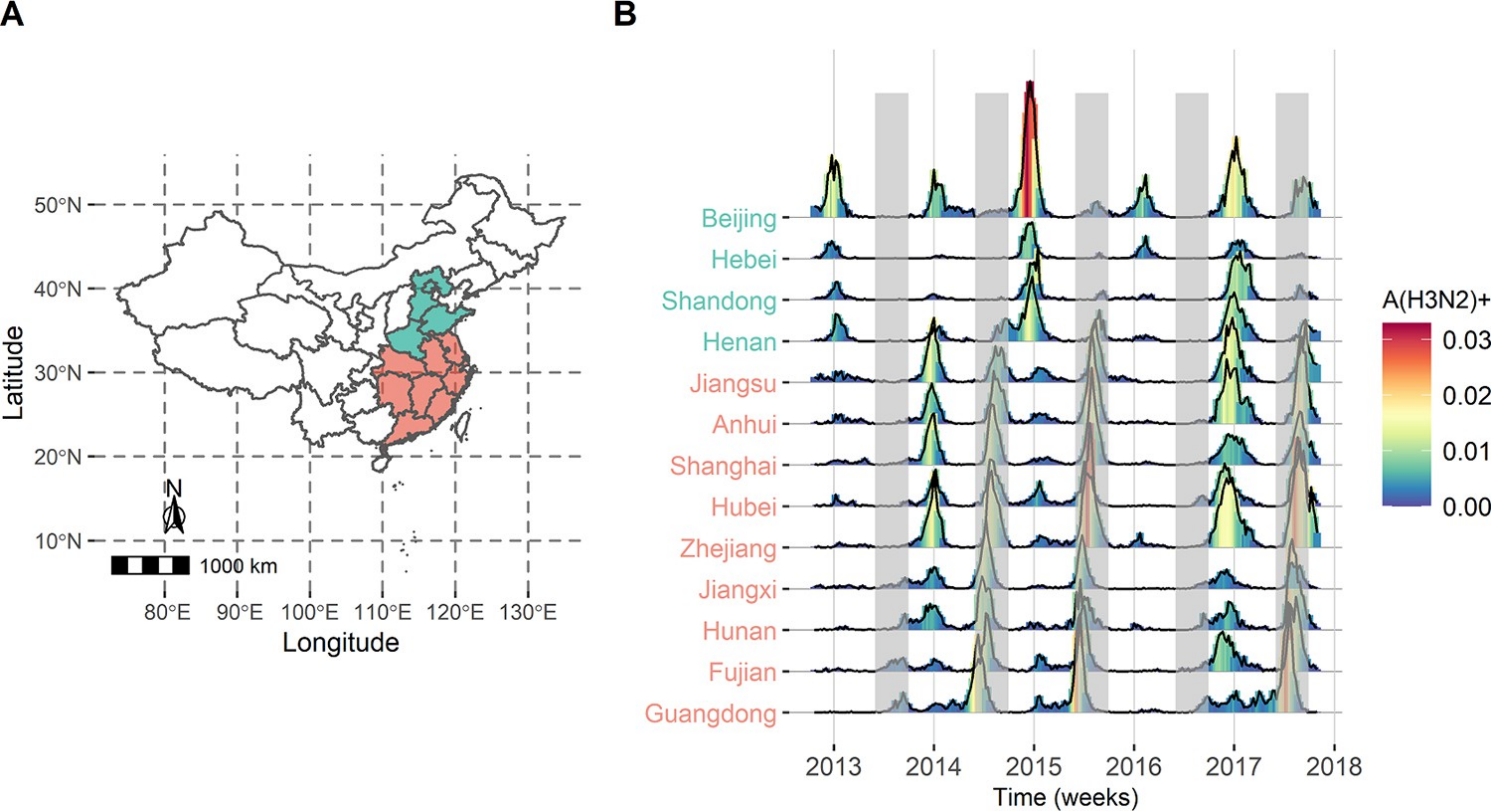
Dynamics of influenza A(H3N2) in the selected thirteen regions of China. A) Geographical distribution of the selected regions that include nine from the southern region (filled in red) and four from the northern region (filled in green). B) Time-series of the proxy incidence rate for influenza A(H3N2) (A(H3N2)+) in the selected regions between 2012 and 2018. The grey shadows represent the summer/autumn months (June, July, August and September) in China. Map was made using R Software (https://www.r-project.org). Source of the basemap shapefile: National Catalogue Service For Geographic Information (https://www.webmap.cn/main.do?method=index).

Thirteen regions (provinces or municipalities) that lie in the comparatively developed areas with a better surveillance system are included in this study, including nine in the southern region of China with obvious summer epidemics of influenza A(H3N2) and four in the northern region of China without the summer epidemic (**Fig 1**). The distribution of absolute humidity shows a clear pattern of lower values in the northern provinces and higher values in the southern provinces (**S1 Fig**). During the study period, the dominant influenza subtype in the winter epidemics varies across influenza seasons, whereas influenza A(H3N2) is the only subtype that is responsible for the summer epidemics (2014, 2015, and 2017) (**S2 Fig**). The seasonal pattern of influenza A(H3N2) varies across regions, with winter epidemics in northern China (e.g., Beijing), bimodal pattern in the middle region of China (e.g., Zhejiang), and stronger summer epidemics in southern China (e.g., Guangdong) (**Fig 1**). For these regions with the obvious summer epidemics, the circulation pattern of influenza A(H3N2) also varies across influenza seasons (**Fig 1**). The winter and summer epidemics coexist in 2013/2014 and 2016/2017, while the winter epidemic is negligible in 2014/2015. Besides, the mean onset week in the winter epidemic in 2016/2017 is approximately 5 weeks earlier, but the end week in the summer epidemic is delayed (**S3 Fig**), compared with the 2013/2014 and 2014/2015 seasons.

### Meta-population model incorporating climatic driver and antigenic change

To examine which biological processes contribute to the observed dynamics of influenza A(H3N2) in China, a meta-population model incorporating the climatic driver and antigenic change was constructed for these three influenza seasons (2013/2014, 2014/2015, and 2016/2017) (**see Materials and Methods**). Results from the model validation on the synthetic time-series show that the multiscale transmission model can accurately re-estimate the key parameters (**S4 Fig**), even when the pre-defined relationship between absolute humidity and influenza A(H3N2) transmissibility varies with different shapes (**Check S1 Text for detail: Model validation for the meta-population transmission model**). When fitting to the real surveillance data, the model can successfully recover the distinct patterns of influenza A(H3N2) dynamics across regions (**S5–S7 Figs**). As a general result, the model that incorporates a punctuated change in the loss of immunity performs better than the one in the transmission process, and both models are significantly better than the one that does not incorporate a change in viral properties (**S2 Table**, *p*<0.05, *likelihood ratio test*). Given the bimodal pattern of influenza A(H3N2) incidence in one influenza season (2013/2014 and 2016/2017), the model with surges of immunity loss both in the winter epidemic and summer epidemic is also constructed, however, the model does not perform significantly better (**S2 Table**). As a result, the model with a single change in immunity waning is used in the following analysis.

The maximum likelihood estimations for the key parameters are given in **Table 1**. Results show that the relationship between absolute humidity and transmission of influenza A(H3N2) exhibits a similar V-shaped one in 2013/2014 (*ω*_0_ = 0.035, 95% Confident Interval (CI): 0.022~0.056; *ω*_1_ = 0.019, 95% CI: 0.011~0.026), 2014/2015 (*ω*_0_ = 0.038, 95% CI: 0.016~0.061; *ω*_1_ = 0.034, 95% CI: 0.029~0.042), and 2016/2017 (*ω*_0_ = 0.003, 95% CI: 0.001~0.005; *ω*_1_ = 0.028, 95% CI: 0.026~0.031) (**S8 Fig**). The climatic dependency for influenza A(H3N2) varies across seasons, but with consistent lowest transmission occurring at an absolute humidity around 12g/m^3^ (*AH*_0_, **Table 1**).

**Table 1:** Maximum likelihood estimations for the key parameters (mean: 95% CI).

Parameters	Influenza seasons		
	2013/2014	2014/2015	2016/2017
ϵ	0.04: 0.02~0.06	0.03: 0.01~0.07	0.05: 0.04~0.06
*AH*_0_(g/m^3^)	11.15: 9.83~12.74	11.19: 10.12~12.19	13.06: 12.17~13.74
*β*_0_	1.19: 1.13~1.25	1.13: 1.10~1.19	1.16: 1.15~1.17
*ω*_0_	0.04: 0.02~0.06	0.04: 0.02~0.06	0.00: 0.00~0.01
*ω*_1_	0.02: 0.01~0.03	0.03: 0.03~0.04	0.03: 0.03~0.03
*t*_*c*_	22 Jan 2014: 15 Jan 2014 ~ 30 Jan 2014	06 Feb 2015: 26 Jan 2015 ~ 21 Feb 2015	11 Aug 2016: 31 Jul 2016 ~ 18 Aug 2016
*δ*_*ca*_	13.9: 10.88~18.26	8.92: 7.82~10.30	6.05: 5.77~6.30
1/*ξ*′(years)	0.06: 0.05~0.08	0.12: 0.09~0.18	0.06: 0.05~0.07
G	75: 40~180	71: 46~170	91: 45~114

ϵ: The amplitude of the holiday effect

*AH*_0_: The threshold value for AH where the lowest seasonal transmission rate is observed

*β*_0_: The lowest seasonal transmission rate of A(H3N2) virus

*ω*_0_: The coefficient of absolute humidity (AH) on A(H3N2) transmission when AH is smaller than *AH*_0_

*ω*_1_: The coefficient of absolute humidity (AH) on A(H3N2) transmission when AH is larger than *AH*_0_

*t*_*c*_: The occurring time when the properties of A(H3N2) virus changed

*δ*_*ca*_: The effect of changes in viral properties on immunity waning

*ξ*′: The rate of immunity waning that population infected by other subtypes lose their immunity to A(H3N2)

G: The gravitation constant in the meta-population model

On the other hand, the occurring times of antigenic change are estimated to be in the winter-spring months for 2013/2014 (22 Jan 2014, 95% CI: 15 Jan 2014 ~ 30 Jan 2014) and 2014/2015 (06 Feb 2015, 95% CI: 26 Jan 2015 ~ 21 Feb 2015), however, at summer-autumn months for 2016/2017 (11 Aug 2016, 95% CI: 31 Jul 2016 ~ 18 Aug 2016) (**Table 1**). Interestingly, the emergence time of antigenic change, is consistent with the estimated divergent time for dominant strains for each influenza season (**S9 Fig**). The rate of immunity waning due to antigenic change increased by 13.90 (95% CI: 10.88~18.26) times for 2013/2014, 8.92(95% CI: 7.82~10.30) times for 2014/2015 and 5.98 (95% CI: 5.70~6.25) times for 2016/2017, respectively, resulting in a shortened recovery period of 0.43 years (95%CI:0.33~0.55) for 2013/2014, 0.67 years (95%CI:0.58~0.67) for 2014/2015, and 1.00 years (95%CI:0.96~1.05) for 2016/2017. The intersubtypic cross-protection from other influenza subtypes to A(H3N2) (*ξ*′, **see Methods and Materials**) also varied in these three flu seasons. The duration that other subtypes of infections lose their immunity to A(H3N2) virus was estimated at 0.06 years (95%CI: 0.05~0.08) for 2013/2014, 0.12 years (95%CI:0.09~0.18) for 2014/2015 and 0.06 years (95%CI: 0.05~0.07) for 2016/2017, respectively.

### Joint effects of population susceptibility, climatic factors, and antigenic change

Since the average of the estimated value for ϵ is 0.04 (**Table 1**), the estimated *β*_*h*_(*t*) during the school term (*β*_*h*_(*t*) = 1.01) and holiday term (*β*_*h*_(*t*) = 0.96) is close (**Eq 5**). Besides, removing holiday effects from the model had a much weaker impact on the model fit than removing the effect of environmental factors and antigenic change (**S3 Table**). Therefore, only the joint effects of initial population susceptibility (population susceptibility at the onset time), climatic factors, and antigenic change are further discussed (**Check S1 Text for detail: Contribution of driving factors on the dynamics of influenza A(H3N2)**). Results from the designed simulations (**Table 2**) show that these three driving factors explain an average of 55% of variations in the dynamics of influenza A(H3N2). Specifically, when the effects of the other two factors are maintained at the estimated levels, the additional variation explained by the initial population susceptibility, climatic factors, and antigenic change are at 33%, 26%, and 48%, respectively. The effect of any single factor on the dynamics of influenza A(H3N2) is limited, with the explained variations less than 10%.

**Table 2:** Variations explained by the major driving factors on the dynamics of influenza A(H3N2).

Influenza seasons	Initial population susceptibility	Antigenic change	Climatic factors	Coefficient of determination
2013/2014	×	×	×	0.08
	×	×	√	0.08
	×	√	×	0.11
	√	×	×	0.14
	×	√	√	0.27
	√	×	√	0.24
	√	√	×	0.17
	√	√	√	0.56
2014/2015	×	×	×	0.11
	×	×	√	0.08
	×	√	×	0.12
	√	×	×	0.15
	×	√	√	0.36
	√	×	√	0.10
	√	√	×	0.34
	√	√	√	0.71
2016/2017	×	×	×	0.06
	×	×	√	0.08
	×	√	×	0.19
	√	×	×	0.07
	×	√	√	0.48
	√	×	√	0.10
	√	√	×	0.38
	√	√	√	0.60

**Fig 2:**
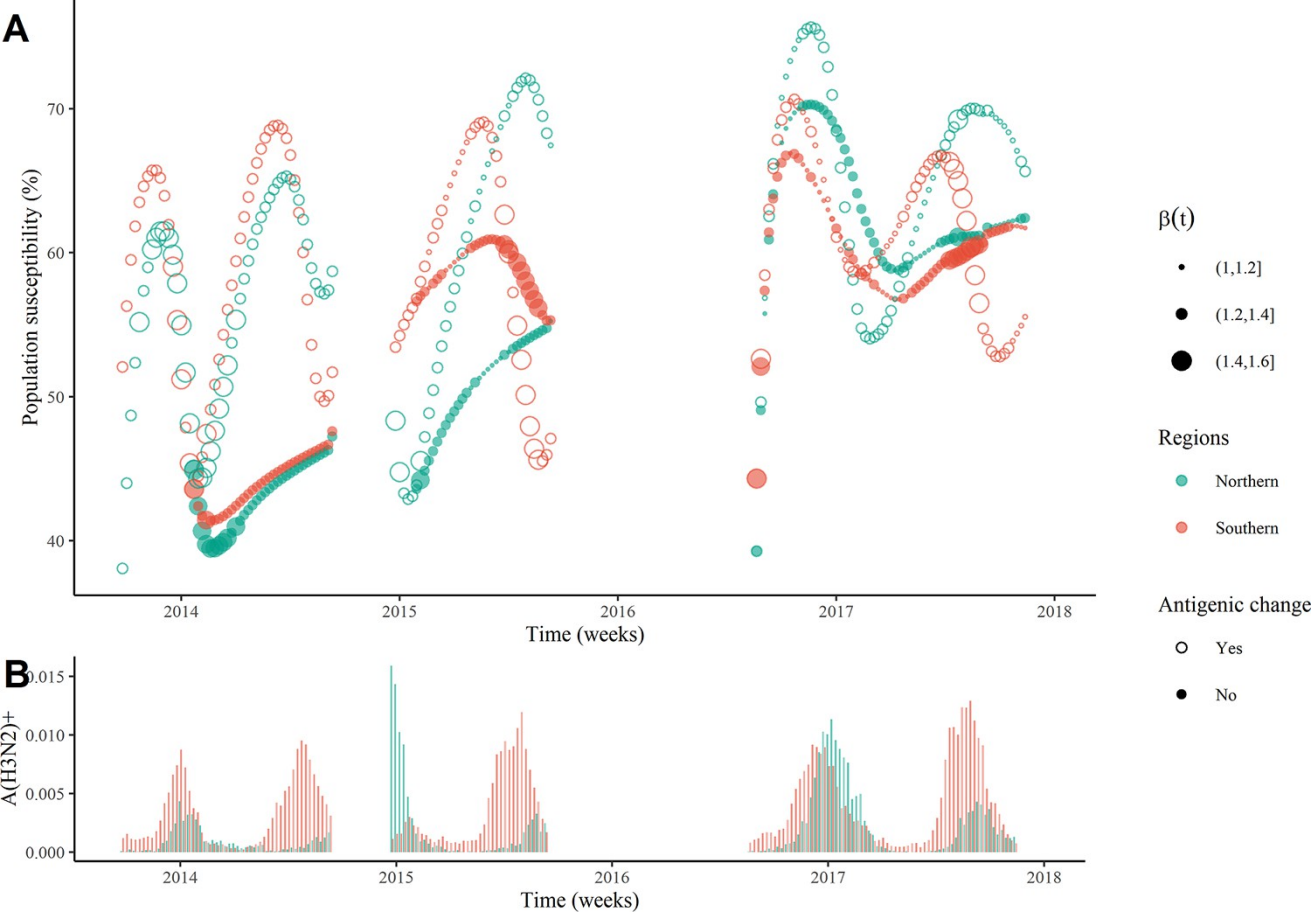
**Dynamic changes of population susceptibility, seasonal transmission rates (A) and incidence of influenza A(H3N2) (B) in the selected northern (green) and southern (red) regions of China.** Based on the maximum likelihood estimations in the meta-population model for the 2013/2014, 2014/2015, and 2016/2017 influenza seasons, the population susceptibility under two scenarios (hollow circles for the situation with the antigenic change and solid circles for the one without the antigenic change) were obtained. The size of points in the top panel represents the average of estimated seasonal transmission rates (*β*(*t*)) for influenza A(H3N2) across regions.

A quantitative description of the dynamics of influenza A(H3N2) provides a better understanding of the interplay between the major driving factors (**Figs 2, S10 and S11**). At the onset time of the winter epidemic in 2013/2014, the proportion of susceptible population are at 40.0% (range: 27.9 ~55.6%) for the northern regions and 52.1% (range: 41.5~65.1%) for the southern regions. The increased population susceptibility due to the waning immunity, combined with the seasonal variations in transmission rates (size of the circle in **Fig 2A**), trigger the winter epidemic (**Fig 2B**). At the end of the winter epidemic, a novel antigenic variant emerges (clade 3C.3a [14]), which accelerates the rate of immunity waning and results in the high level of population susceptibility of 66.4% (range: 62.6~69.9%) for the northern regions and 72.4% (range: 68.2~75.6%) for the southern regions in June of 2014 (hollow circles in **Fig 2A**), compared with 45.8% (range: 44.9~48.1%) for the northern regions and 47.2% (range: 42.2~57.8%) for the southern regions when no antigenic change emerged (solid circles in **Fig 2A**). A similar changing pattern for the population susceptibility is observed in 2014/2015 (due to the clade 3C.2a [7]), with the comparable level of population susceptibility in the northern and southern regions at June of 2015. Due to the lower seasonal transmission rates between March and May in the northern regions compared to that in the southern regions (size of the circle in **Fig 2A**), an obvious summer epidemic occurs in the southern regions rather than the northern regions (**Fig 2B**). Interestingly, for the 2016/2017 season, the population susceptibility is at 39.2% (30.3~53.1%) for the northern regions and 44.3% (range: 30.5–53.1%) for the southern regions at August of 2016, then subsequently increases to 75.8% in the northern regions and 71.5% in the southern regions (due to the clade 3C.2a2 [15]), and decreases to a low level at nearly 56.0% because of the susceptible depletions during the winter epidemic. The population susceptibility of nearly 65% in the southern regions at June of 2017 is comparable to that in the northern regions, however, in the former, high seasonal transmission rates (>1.3) of influenza A(H3N2) appear earlier and persist for a longer time (**Fig 2A**), ultimately trigger the summer epidemic. In the northern regions, the increased population susceptibility of around 70% in August of 2017, compensates for the effect of the relatively unsuitable climatic conditions for A(H3N2) transmission, and eventually leads to a less intense epidemic of influenza A(H3N2) (**Fig 2B**).

### Effects of interventions on the summer epidemics of A(H3N2)

**Fig 3:**
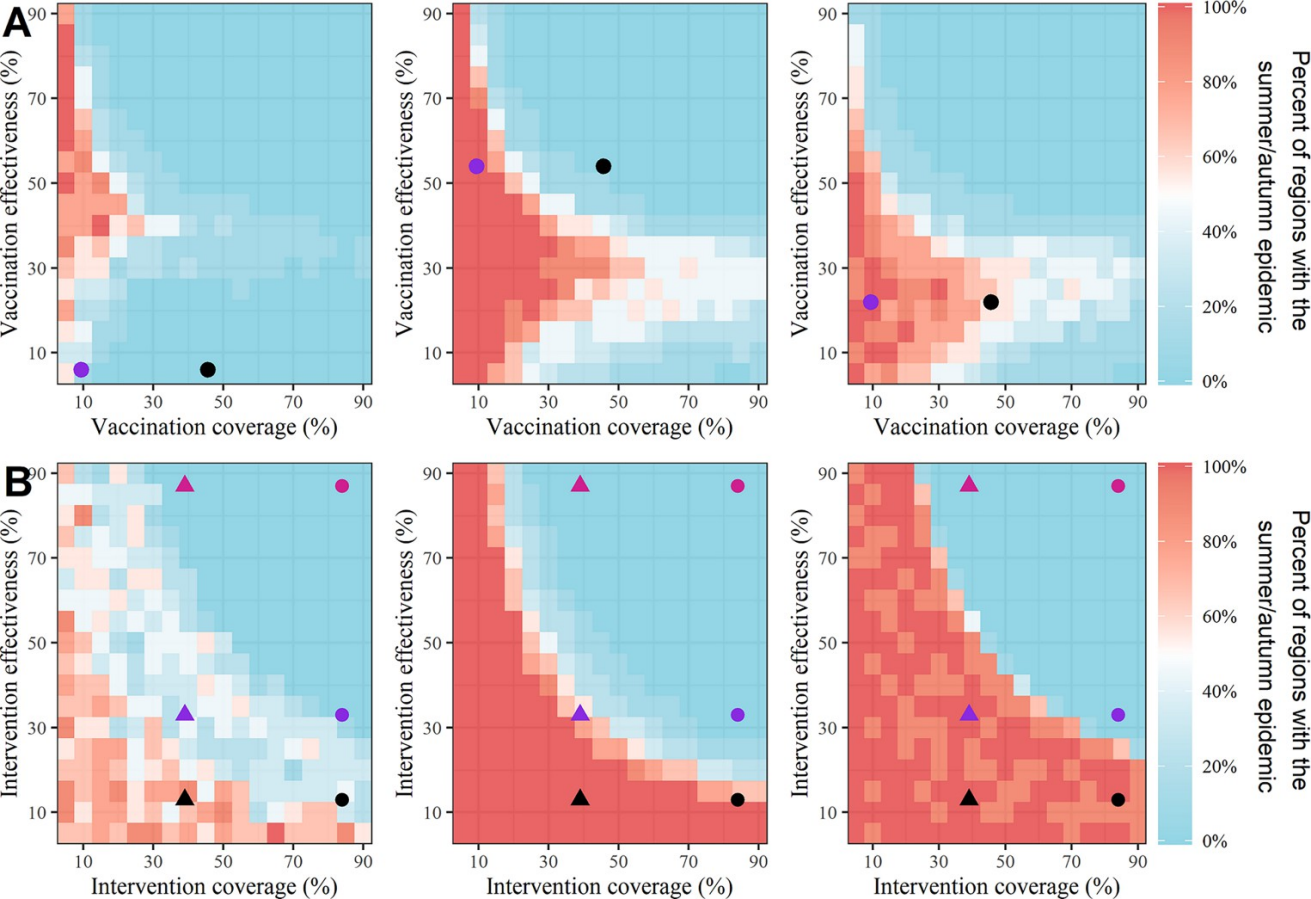
**The effects of pharmaceutical intervention (vaccination program, A) and non-pharmaceutical intervention (using facemasks plus hand hygiene, B) on the summer epidemics of influenza A(H3N2) in 2013/2014 (Left), 2014/2015 (Middle) and 2016/2017 (Right)**. The triangles and circles in the top panel represent the influenza coverage in China (9.4% [19]) and United States (43.8% [20]), respectively; while the color represents the average (black, 29%) and season-specified vaccine effectiveness (blue,0.09 for 2014/2015 [16], 0.54 for 2015/2016 [17] and 0.22 for 2017/2018 [18]). The triangles and circles in the bottom panel show the percentage of the population using facemasks plus hand hygiene in the non-pandemic period (39% [22]) and pandemic period (84% [23]), respectively. The color in the triangles or circles represents the low (13%, black), middle (23%, blue), and high (87.0%, red) effectiveness of using facemasks plus hand hygiene to prevent influenza A(H3N2) [21], respectively.

Because the summer epidemic of influenza A(H3N2) is more fragile compared to the winter epidemic and there is no obvious summer epidemic in the northern region of China, the effects of the pharmaceutical intervention (vaccination program) and non-pharmaceutical intervention (using facemasks plus hand hygiene) are only tested on the summer epidemic (**Check S1 Text for detail: Effects of interventions on the summer epidemics**). Results show that, for those three influenza seasons, when the vaccine effectiveness is at the known level (0.09 for 2014/2015 [16], 0.54 for 2015/2016 [17] and 0.22 for 2017/2018 [18], and the vaccine effectiveness in the summer epidemic is assumed based on the estimated vaccine effectiveness in the following influenza season), the low vaccination coverage for influenza in China (9.4% [19]) will fail to prevent the summer epidemic of influenza A(H3N2) (**Fig 3A**). If the vaccine coverage maintains at the high level as similar to that in the United States (43.8% [20]), the summer epidemic of influenza A(H3N2) could be prevented in 2013/2014 and 2014/2015, but not in 2016/2017 (blue circles in **Fig 3A**). Nevertheless, nearly 14.3, 89.2, and 39.6 percent of A(H3N2) infections in the summer-autumn months could be prevented by the vaccination program in those three influenza seasons (**S12A Fig**). The vaccine mismatch for influenza A(H3N2) in 2013/2014 is obvious [16], and if the vaccine effectiveness can be improved to the mean level (0.29), the number of regions that experiencing the summer epidemic would not decrease (**Fig 3A**), but the percent of A(H3N2) infections prevented by the vaccination program could be increased to 31% (**S12A Fig**).

It is worth noting that, the summer epidemics of influenza A(H3N2) can also be mitigated by non-pharmaceutical interventions (**Fig 3B**). For instance, when the effectiveness of using facemasks plus hand hygiene to prevent A(H3N2) infections is at 0.33 [21], the population coverage of using facemasks plus hand hygiene needs to be increased to around 70% in these three influenza seasons. Interestingly, this threshold is larger than the coverage of 39% in the non-pandemic period [22], but smaller than that of 84% in the coronavirus pandemic period [23] (**Fig 3B**). The intervention coverage is only required to be around 30% when the effectiveness of using facemasks plus hand hygiene is at 87% [21]. Even when the effectiveness of using facemasks plus hand hygiene to prevent A(H3N2) infection is low at 0.13 [21], the intensity of influenza A(H3N2) in the summer-autumn months of 2013/2014 can be reduced to the low level (A(H3N2)+ less than 0.005) (**S12B Fig**).

## Discussion

To quantitatively understand which driving factors and how they impact the spread of influenza A(H3N2) virus, requires careful exploration of both the temporal and spatial heterogeneity in the surveillance data with a suitable integrated framework. However, combining multiple driving factors in a modeling framework presents several theoretical challenges. In the present study, an AH-driven meta-population model based on the multi-source data (climatic, demographic, virological and epidemiological data) is constructed for three influenza seasons where obvious summer epidemics of influenza A(H3N2) exist. Compared with the prior studies that directly fit the long-term surveillance data [2,6], our study treats each season independently, to diminish the impact of variability in immunity propagation on influenza dynamics across influenza seasons [5,24]. Although the selected time series in each influenza season is relatively short, the heterogeneity of influenza A(H3N2) seasonality (**Fig 1**) as well as the diverse climate across regions (**S1 Fig**), provide a blessed opportunity to gain insights into the underlying mechanism for the circulation of influenza A(H3N2). Besides, compared with the transmission model of a single region, the spatially structured model is of great utility in parameter identifiability, particularly when process noise and observation error are large [25,26]. The integrated framework present here, can also be applied to obtain novel insights into the impact of driving factors on other infectious diseases.

The antigenic change, as one of the most important driving factors [2,5], is essential for the summer epidemics of influenza A(H3N2) in China (**Fig 2**). In the present study, the largest rate of immunity waning is observed in 2013/2014 (**Table 1**), which is consistent with the fact that the dominant clade 3C.3a in this influenza season is antigenically drifted from the vaccine strain A/Victoria/ /361/2011 [14]. Besides, the occurring times of novel antigenic variants estimated from the meta-population model are consistent with the results from the phylogenetic analysis (**S9 Fig**), suggesting that the molecular footprint of the influenza virus can provide valuable information for modelling the influenza dynamics. Meanwhile, three antigenic changes are observed in four consecutive influenza seasons in China. Such frequent antigenic changes complicated the dynamics of influenza A(H3N2) and resulted in an intriguing biannual pattern of influenza A(H3N2) epidemics in the temperate regions (e.g., Yangtze River Delta Region, **Fig 1**). On the other hand, due to the weak antigenic variability between 3C.2a2 (the dominant clade since 2016), 3C.2a (the dominant clade in 2015) and other subclades (3C.2a1, 3C.2a3, and 3C.2a4) [15], the emergence of new antigenic variants of A(H3N2) virus co-circulated with, rather than completely replaced, the old one since 2015 [7]. This co-circulation pattern of antigenic variants not only complicated the dynamics of influenza A(H3N2) but also posed a great challenge for the selection of appropriate candidate vaccine strains from circulating strains. The renewed source of the susceptible population who lost the heterosubtypic immunity, also fueled the summer epidemics of influenza A(H3N2). It was estimated that the heterosubtypic infection elicited a short-term cross-immunity (small than one month, **Table 1**) to A(H3N2) virus, enforcing a large number of the population infected by the B-lineage or and A(H1N1)pdm09 in the winter epidemics into the susceptible population again. Both epidemiological and laboratory studies have shown that pre-existing immunity with B-lineage did not block A(H3N2) virus transmission [27,28]. Therefore, it is not surprising that weak cross-immunity between subtypes was observed in this study, since the dominant strain was B-lineage in the winter epidemics in these three influenza seasons (**S2 Fig**).

Seasonal variation in the dynamics of influenza A(H3N2) is among the oldest observations in population biology, yet our understanding of the mechanisms underlying this phenomenon remains hazy at best [29]. Both the epidemiological and experimental studies highlight the importance of absolute humidity on influenza seasonality [8,11,30]. However, most of the previous studies focused on the climatic dependence of influenza but not directly on influenza A(H3N2) [9,11]. In this study, after adjusting the effect of antigenic change on immunity waning and the holiday effect on transmission, we find that there is a nonlinear relationship between absolute humidity and A(H3N2) transmission, with the lowest transmission at 12g/m^3^ of absolute humidity, which is consistent with the results from the previous studies based on the global data [10,11]. Surprisingly, the climatic dependence varies among influenza seasons (**S8 Fig**). The slightly different shape for the dependence of transmission on absolute humidity could be explained by several plausible alternative hypotheses. The segmentation function used in this study may not fully capture the nonlinear relationship between AH and influenza transmission [31]. Besides, since other environmental factors that have an impact on influenza transmission are not incorporated into the model structure, the relationship between AH and influenza transmission may be modified to better fit the surveillance data. Given the fact that the anomalous dynamics (earliest start and longer duration) of influenza A(H3N2) in 2016/2017 were not only observed in China (**S3 Fig**), but also in other regions such as Europe [32], we speculate that the mutations have altered the environmental adaptation of influenza virus and allowed them to be prevalent in an anomalous way. Although evidence about the AH-related mutations lacks for influenza A(H3N2), studies have already confirmed that specific mutations in influenza virus are responsible for controlling the temperature-related phenotypes (i.e, temperature-sensitive and cold-adapted mutations) [33–35]. It seems that viruses have an incredible capacity to adapt to environmental challenges, but sometimes, the ambient environment constrains viral adaptation [36]. Nevertheless, more evidence, including both laboratory and epidemiological, is needed to discover the molecular mechanism for the environment-related fitness of the influenza A(H3N2) virus.

Influenza vaccination is the primary strategy to prevent influenza infection and its complications, especially for elderly people [18,37]. The marked geographic differences in influenza seasonality pose a great challenge to specify an uniform influenza vaccination strategy in China [4]. Since influenza A(H3N2) is the only subtype responsible for the summer epidemic (**S2 Fig**), elimination of the summer epidemics is of great importance not only in reducing the disease burden, but also as a test to establish the framework for policy evaluation to help for optimizing the routine influenza immunization campaigns. Given the fact that influenza vaccination coverage is low in China and vaccine effectiveness varies with the matching of vaccine strains to circulating strains [19], relying solely on the vaccination program would be not sufficient to suppress the summer circulations (**Fig 3**). Nevertheless, even imperfect vaccines may be of great benefit because increasing the proportion of vaccinated individuals can supply enough herd immunity and contribute a lower disease burden [38]. Using facemasks plus hand hygiene, as one of the non-pharmaceutical interventions that are easily applicable and accessible for the general population, has been highly recommended to suppress the spreading of seasonal influenza [21]. The model simulation indicates that when the population coverage for using facemasks plus hand hygiene was maintained at the same level during the coronavirus pandemic period, the summer epidemics of influenza A(H3N2) could be prevented (**Fig 3**). This is consistent with the low incidence rate of A(H3N2) in China during the 2020/2021 influenza season [39], as facemasks were required on all public transport.

Multiple limitations should be acknowledged. First, environmental factors such as temperature and ultraviolet radiation were also associated with A(H3N2) transmission [9], however, only absolute humidity was used as seasonal forcing in the present study. Further studies, which take these environmental variables into account, will need to be undertaken [31]. Secondly, although multi-source data have been fed into an integrated framework, other biological processes (such as population heterogenicity) may be involved in the influenza dynamics, leading to the intricate and diverse transmission pattern across regions [5,40]. Thirdly, due to the lack of detailed data on population mobility in China, we used a gravity model with the basic form to measure the number of travelers. More complicated forms with additional tuning parameters or/and combined with real-world data may be more realistic. Finally, the kinetic model was constructed for multiple regions within a single influenza season and therefore becomes complex and computationally demanding when dealing with long time series. Future work will optimize the model framework so that it can reveal the influenza dynamics over a long time series. Nevertheless, this is the first attempt to tease out the important drivers behind the complicated transmission pattern of seasonal influenza A(H3N2) in China, which could shed light on the precise prevention and control of seasonal influenza and other important infectious diseases.

## Materials and methods

### Data

Provincial-level outpatient influenza-like illness (ILI) surveillance data and viral surveillance data were obtained from the Chinese National Influenza Center, National Institute for Viral Disease Control and Prevention. Pandemic influenza H1N1 virus (A(H1N1)pdm09) and two lineages of influenza B virus (B-lineage) were combined. The subtype-specific proxy incidence rate (henceforth termed A(H3N2)+ for influenza A(H3N2) and influenza+ for either influenza A(H3N2), A(H1N1)pdm09 or B-lineage) was calculated as the product of ILI rate per consultations and the proportion of ILI samples that tested positive for influenza subtype. A summer epidemic (winter epidemic) is defined as one in which both the peak week occurred in the summer-autumn months (winter-spring months) and the peak intensity for A(H3N2)+ is greater than 0.005. The summer-autumn months are defined as June, July, August, and September, while the winter-spring months are defined as November, December, January and February. For each influenza season, the origin period was defined as October to September of the following year. The study period in each influenza season was confined to the epidemic period, which ranges from the latest onset week in the winter epidemic to the latest ending week in the summer epidemic across regions. The onset week was defined as the first week of at least 3 consecutive weeks with a non-zero A(H3N2)+, and the ending week is the last week of at least 3 consecutive weeks with the non-zero A(H3N2)+ [41]. Yearly provincial-level population and birth population were gathered from the China Statistical Yearbook published in the National Bureau of Statistics of China (http://www.stats.gov.cn/). Daily absolute humidity (AH) in each province was downloaded from the China Meteorological Data Sharing Service System (http://cdc.cma.gov.cn/home.do). Genome sequences of the human seasonal influenza A(H3N2) virus were collected from the Global Initiative on Sharing All Influenza Data (https://www.gisaid.org).

### Meta-population transmission model

Previous studies suggested that the dynamics of influenza A(H3N2) were shaped by multiple driving factors, including antigenic change, climatic factors, human behavior, population susceptibility and competition among influenza subtypes [2,5,6,42]. As a result, an AH-driven meta-population model that incorporates those major influencing factors was constructed for influenza A(H3N2) in each influenza season. A modified susceptible-infected-recovery-susceptible (SIRS) model was utilized here [2], which divided the whole population into the susceptible (S), the infected (I), the recovered (R) classes, and a class for the recovery population infected by either influenza A(H1N1)pdm09 or B-lineage (*R*′). Considering the low vaccination coverage of seasonal influenza in China (9.4% among the general population [19]), the model used here has no compartment explicitly representing the vaccinated population. The detailed differential equations were formulated as follows:

$$\frac{dS_{i}}{dt}=B_{i}-\lambda_{i}(t)S_{i}+\xi (t)R_{i}-\frac{\Lambda_{i,t}}{\rho_{i}^{\prime }}+R_{i}^{\prime }\xi^{\prime }-\mu S_{i}$$

$$\frac{dI_{i}}{dt}=\lambda_{i}(t)S_{i}-\gamma I_{i}-\mu I_{i}$$

$$\frac{dR_{i}}{dt}=\gamma I_{i}-\xi (t)R_{i}-\mu R_{i}$$ (1)

$$\frac{dR_{i}^{\prime }}{dt}=\frac{\Lambda_{i}}{\rho_{i}^{\prime }}-R_{i}^{\prime }\xi^{\prime }-\mu R_{i}^{\prime }$$

$$N_{i}=S_{i}+I_{i}+R_{i}+R_{i}^{\prime }$$

where *B*_*i*_ and *N*_*i*_ denote the birth and total population for region *i*, respectively. *μ* is the death rate. 1/*γ* and *ξ*(*t*) are the infectious period and the rate of immunity waning for the population infected by influenza A(H3N2). Λ_*i*,*t*_ denotes the new observed cases of other influenza subtypes at the time-point *t* for region *i*, which was designed to track the reduction in the population susceptibility due to the infections of other influenza subtypes [2]. On the contrary, the recovery population infected by either A(H1N1)dpm09 or B-lineage (*R*′) would be susceptible to influenza A(H3N2) by the rate of *ξ*′, due to the partial cross-immunity among influenza subtypes [28]. Given the geographical heterogeneity in disease reporting, the average reporting probability ($\rho_{i}^{\prime }$) for the infections by either A(H1N1)dpm09 or B-lineage is formulated as $\rho_{i}^{\prime }=\rho_{0}^{\prime }*\upsilon_{i}$, where $\rho_{0}^{\prime }$ is the basic reporting probability and *υ*_*i*_ is the regional scale factor.

To simplify the model structure, it is assumed that the population migration among regions was symmetric, thus, the force of infection (*λ*_*i*_(*t*)) for influenza A(H3N2) in the region *i* can be formulated as follows:

$$\lambda_{i}(t)=\beta_{i}(t)\left[\frac{I_{i}}{N_{i}}+\sum_{k\neq i}\frac{v_{ik}}{N_{i}}(\frac{I_{k}}{N_{k}}-\frac{I_{i}}{N_{i}})\right]$$ (2)

where *v*_*ik*_ is the population flow from region *i* to region *k*. Due to the inaccessibility of the cross-provincial population migration data in China, a gravity model with the basic form is utilized here to describe the number of travelers [43]:

$$v_{ik}=G*\frac{\bar{d}}{{\bar{N}}^{2}}*\frac{N_{i}N_{k}}{d_{ik}}$$ (3)

where *d*_*ik*_ denotes the distance between region *i* and region *k*. The gravitation constant G is scaled to the average population $\bar{N}$ of all regions and their average distance $\bar{d}$.

The time-varying transmission rate *β*_*i*_(*t*) is measured using the product of seasonal transmission rates (*β*_*s*,*i*_(*t*)), holiday effect (*β*_*h*_(*t*)) or/and the effect of viral mutations (*β*_*a*_(*t*)).

### 1) The seasonal transmission rate *β*_*s*,*i*_(*t*)

Given the importance of AH on influenza transmission [8], an AH-driven transmission model was constructed here. Previous studies implied that there was a nearly U-shaped relationship between AH and influenza transmissibility [10], thus, a segmented function was utilized in the study to measure the seasonal transmission rate (*β*_*s*,*i*_(*t*)) for region *i* at the time point *t*:

$$\beta_{s,i}(t)=\left\{ \begin{array}{c} \omega_{0}({AH}_{0}-{AH}_{i,t})+\beta_{0},{AH}_{i,t}<{AH}_{0}\\ \omega_{1}({AH}_{i,t}-{AH}_{0})+\beta_{0},{AH}_{i,t}\geq {AH}_{0} \end{array}\right.$$ (4)

where *ω*_0_ and *ω*_1_ are the coefficient of AH on influenza transmission when *AH*_*i*,*t*_ is smaller or larger than *AH*_0_, respectively. *AH*_0_ is the threshold value for AH where the lowest seasonal transmission rate (*β*_0_) is observed.

### 2) Holiday effect on influenza transmission *β*_*h*_(*t*)

Similar to the previous study [44], the holiday effect on the transmission of influenza A(H3N2) is assumed to be high during the school term and low at the holiday term. In this manner, the equation for *β*_*h*_(*t*) can be written as follows:

$$\beta_{h}(t)=\left\{ \begin{array}{c} 1+\epsilon *(1-p)/p,during\;the\;school\;term\\ 1-\epsilon ,during\;the\;holiday\;term \end{array}\right.$$ (5)

where *p = 0*.*77* is the proportion of the year taken up by the school term, and *ϵ* is the amplitude of the holiday effect on influenza transmission. The calendar-day timings of the school holidays are set as follows: New Year’s Day (1–3), Winter Holiday (18–50), International Labor Day (121–123), Summer Holiday (212–273), and National Day (240–247).

### The effect of viral mutations

In this study, we explicitly included the effect of viral mutations in the meta-population model, a property critical for the dynamics of influenza A(H3N2). Two hypothesized scenarios were tested for each influenza season: i) mutations associated with increased transmissibility, ii) mutations associated with antigenic change that leads to the loss of protection from prior infection [2,45]. To simplify the model structure, an unified occurring time (*t*_*c*_) and the effect of viral mutations (*δ*_*ct*_ or *δ*_*ca*_) are utilized for all the selected regions.

The change in transmissibility due to viral mutations is modeled as follows:

$$\beta_{a}(t)=\left\{ \begin{array}{c} 1,t<t_{c}\\ \delta_{ct},t\geq t_{c} \end{array}\right.$$ (6)

And the change in immunity waning due to antigenic change is modeled as follows:

$$\xi (t)=\left\{ \begin{array}{c} \xi_{0},t<t_{c}\\ \xi_{0}\delta_{ca},t\geq t_{c} \end{array}\right.$$ (7)

In the second scenario, the new recovery period with the impact of antigenic changes was denoted by the value of 1/(*ξ*_0_*δ*_*ca*_).

To explore whether the antigenic change of influenza A(H3N2) exists and which scenario is more reasonable to represent the effect of antigenic change on the dynamics of influenza, likelihood-based criteria were used for model selection, including a likelihood ratio test when the models are nested and the Akaike information criterion, which penalizes the likelihood based on the number of parameters [46].

### Inference framework

In the observation process, the number of weekly cases, *Y*_*i*_(*t*), is given by the cumulative transitions from the susceptible compartment (S) to the infected compartment (I). Thus, the observed number of weekly influenza A(H3N2) cases *C*_*i*_(*t*) is assumed to follow a discretized normal distribution allowing for the over-dispersion on the diseases reporting [44,47]:

$$C_{i}(t)|Y_{i}(t)∼rnormal(\rho_{i}Y_{i}(t),\sigma_{i,t})$$ (8)

where *ρ*_*i*_ is the reporting probability per infection for region *i*. Similar to $\rho_{i}^{\prime }$, *ρ*_*i*_ is formulated as the product of the scale factor (*υ*_*i*_) and basic reporting probability of influenza A(H3N2) infections (*ρ*_0_). *σ*_*i*,*t*_ is the variance in the normal distribution, which is formulated as follows:

$$\sigma_{i,t}=\rho_{i}(1-\rho_{i})Y_{i}(t)+{\left(\varrho \rho_{i}Y_{i}(t)\right)}^{2}$$ (9)

where *ϱ* is an over-dispersion parameter that defines the measurement error [44].

To reduce the model complexity, the human death rate was fixed at 1/76 per year, reflecting the current life expectancy of China in 2016 (http://www.stats.gov.cn). The infectious period (1/*γ*) and recovery period (1/*ξ*_0_) are set at 3 days and 6 years according to the data from previous studies [28,48,49]. A detailed description of all parameters in the meta-population transmission model was listed in **S1 Table**. The meta-population model was constructed using the R software spatPomp package [47,50]. Parameters were optimized for each influenza season through the sequential Monte Carlo method [51]. To obtain approximate 95% confidence intervals (CI) of the target parameters, the Monte Carlo Adjusted Profile algorithm was used [52].

## Supporting information

S1 Fig Overall distribution (left) and region-specific distribution (right) of absolute humidity during the epidemic period in the 2013/2014 (A), 2014/2015 (B) and 2016/2017 (C) influenza seasons.
(TIF)

S2 Fig The time-series of subtype-specific influenza data (influenza+) in the northern and southern regions of China between 2013 and 2018. The influenza+ was calculated as the product of the influenza-like illness incidence rate, the proportion of influenza-like illness samples that tested positive for influenza and the subtype-specific proportion. Northern regions: Beijing, Hebei, Henan and Shandong. Southern regions: Guangdong, Jiangxi, Hunan, Hubei, Fujian, Zhejiang, Jiangsu, Anhui and Shanghai. The grey shadows represent the summer-autumn months (June, July, August and September) in China.
(TIF)

S3 Fig Dynamics of influenza A(H3N2) in the selected southern regions of China in the 2013/2014, 2014/2015 and 2016/2017 influenza seasons. Seasons were color coded as green, blue and red for 2013/2014, 2014/2015 and 2016/2017 respectively. A) The time-series of A(H3N2)+ for nine selected regions from southern China. B) The relationship between the peak week of the summer epidemics and the latitude in nine southern regions of China. C) The relationship between the peak week of the winter epidemics and the latitude in nine southern regions of China. The most northern region Jiangsu among the selected nine regions was taken as the reference to calculate the weekly difference of peak times in B and C. A linear regression model was utilized here to explore the relationship between the weekly difference of peak times and latitude. The black dashed line in B and C represent the linear fitted line. A significant relationship between the weekly difference of peak times and latitude was observed in the summer epidemics (*p* < 0.01), but not in the winter epidemics (*p* = 0.92).
(TIF)

S4 Fig Model validation for the meta-population transmission model. Four scenarios were tested here with the different relationship between absolute humidity (AH) and the transmission of influenza A(H3N2): the pre-defined values for *ω*_0_ and *ω*_1_ at either high (0.05) or low (0.01). If the value of *ω*_0_ and *ω*_1_ were set at the low and high values, respectively, the relationship between AH and A(H3N2) transmission was formulated as the “J” relationship. Other parameters and initial states were obtained from the maximum likelihood estimations in the meta-population transmission model for the influenza season 2013/2014. Based on the simulated time-series, the maximum likelihood estimation and its 95% confidence interval for the key parameters (black dots and lines in the figure) was re-estimated based on the inference framework and compared with the pre-defined value (red dots).
(TIF)

S5 Fig Model fitting on the surveillance data of A(H3N2) in the 2013/2014 influenza season. In total, thirteen regions were selected, including four regions (Beijing, Hebei, Shandong and Henan) without the obvious summer epidemics of influenza A(H3N2) virus. The black line represents the surveillance data, while the red one shows the median value of the simulated time-series based on the maximum likelihood estimation in the meta-population model with the 95% confidence interval (red shadows).
(TIF)

S6 Fig Model fitting on the surveillance data of A(H3N2) in the 2014/2015 influenza season. In total, thirteen regions were selected, including four regions (Beijing, Hebei, Shandong and Henan) without the obvious summer epidemics of influenza A(H3N2) virus. The black line represents the surveillance data, while the red one shows the median value of the simulated time-series based on the maximum likelihood estimation in the meta-population transmission model with the 95% confidence interval (red shadows).
(TIF)

S7 Fig Model fitting on the surveillance data of A(H3N2) in the 2016/2017 influenza season. In total, thirteen regions were selected, including four regions (Beijing, Hebei, Shandong and Henan) without the obvious summer epidemics of influenza A(H3N2) virus. The black line represents the surveillance data, while the red one shows the median value of the simulated time-series based on the maximum likelihood estimation in the meta-population transmission model with the 95% confidence interval (red shadows).
(TIF)

S8 Fig Climatic dependence of influenza A(H3N2) virus in the 2013/2014, 2014/2015, and 2016/2017 influenza seasons. The seasonal transmission rate was nonlinearly formulated using the absolute humidity and four parameters (*R*_0_, *ω*_0_, *ω*_1_ and *AH*_0_) in the meta-population transmission model. Based on the maximum likelihood estimations, the seasonal transmission rates were projected. A) The nonlinear relationship between absolute humidity and the transmission of influenza A(H3N2). B) The geographical heterogeneity in the seasonal transmission rates of influenza A(H3N2). Seasons were color coded as green, blue and red for the 2013/2014, 2014/2015, and 2016/2017 influenza seasons, respectively. Based on the maximum likelihood estimations, the seasonal transmission rates were projected in the 2013/2014(C), 2014/2015(D), and 2016/2017(E) influenza seasons (**see Material and Method**).
(TIF)

S9 Fig Phylogenetic analysis for the hemagglutinin (HA) segment of influenza A(H3N2) virus. A) Bayesian time-scaled phylogenetic tree for influenza A(H3N2) virus in the selected regions of China between 2012 and 2018. Tip strains colored on the phylogenetic tree denote the strains in the summer-autumn months (June, July, August and September) in the 2013/2014 (green), 2014/2015 (brown), and 2016/2017 (red) influenza seasons, respectively. B) Distribution of the divergent times for the HA segment of influenza A(H3N2) virus. The solid lines represent the density distribution of the divergent times for HA of the influenza A(H3N2) virus, while the dashed lines show the estimated occurring times of the antigenic change from the meta-population transmission model (**Table 1).**
(TIF)

S10 Fig Quantitively understanding of population susceptibility on the dynamics of influenza A(H3N2) in the selected northern regions of China. Based on the maximum likelihood estimations in the meta-population transmission model for the 2013/2014, 2014/2015, and 2016/2017 influenza seasons, the propagation of population susceptibility under two scenarios (with or without the antigenic antigenic) was obtained. The black line represents the surveillance data of A(H3N2)+. The red line and shadow represent the median value and its 95% confidence interval of A(H3N2)+ in the simulated time-series with the antigenic change, while the green one for the results without the antigenic change. The right y-axis represents the population susceptibility, which is calculated using the number of susceptible populations dividing the whole population in each region.
(TIF)

S11 Fig Quantitatively understanding of population susceptibility on the dynamics of influenza A(H3N2) in the selected southern regions of China. Based on the maximum likelihood estimations in the meta-population transmission model for the 2013/2014, 2014/2015, and 2016/2017 influenza seasons, the propagation of population susceptibility under two scenarios (with or without antigenic change) was obtained. The black line represents the surveillance data of A(H3N2)+. The red line and shadow represent the median value and its 95% confidence interval of A(H3N2)+ in the simulated time-series with the antigenic change, while the green one is for the results without the antigenic change. The right y-axis represents the population susceptibility, which is calculated using the number of susceptible populations dividing the whole population in each region.
(TIF)

S12 Fig Effects of pharmaceutical intervention (vaccination program) (A) and non-pharmaceutical intervention (using facemasks plus hand hygiene) (B) on the cases of influenza A(H3N2) in the 2013/2014 (left panel), 2014/2015 (middle panel) and 2016/2017 (right panel) influenza seasons. The coverage and effectiveness of interventions range from 5% to 90% with an interval of 5%. The triangles and circles in the top panel (**A**) represent the influenza vaccine coverage in China (9.4% [9]) and United States (43.8% [10]), respectively. Different colors represent the average (black, 29%) and season-specified vaccine effectiveness for influenza A(H3N2) (blue,0.09 for 2014/2015 [11], 0.54 for 2015/2016 [12] and 0.22 for 2017/2018 [13]. The vaccine effectiveness in the summer epidemic was assumed based on the estimated vaccine effectiveness in the following season). The triangles and circles in the bottom panel (**B**) show the percentage of the population using facemasks plus hand hygiene in the non-pandemic period (39% [14]) and pandemic period (84% [15]), respectively, while the color in the triangles or circles represents the low (13%, black), middle (23%, blue) and high (87.0%, red) effectiveness of using facemasks plus hand hygiene to prevent influenza A(H3N2) [16], respectively.
(TIF)

S13 Fig The pipeline for the model validation. (TIF)

S1 Table Description of parameters in the meta-population transmission model. (DOCX)

S2 Table Model tests for the effect of viral mutations on the dynamics of influenza A(H3N2) (Akaike information criterion). (DOCX)

S3 Table Model fitting for the meta-population model under different scenarios (Maximum likelihood value). (DOCX)

S1 Text Supplementary Methods. (DOCX)

S1 File Acknowledgments to the authors and laboratory for registering the genome data via GISAID. (CSV)
