# Supplementary figures and images for: Mechanisms for the circulation of influenza A(H3N2) in China: A spatiotemporal modelling study
Source: PLoS Pathog. 2022 Dec 16;18(12):e1011046. doi: 10.1371/journal.ppat.1011046 (PMC9803318; doi:10.1371/journal.ppat.1011046)

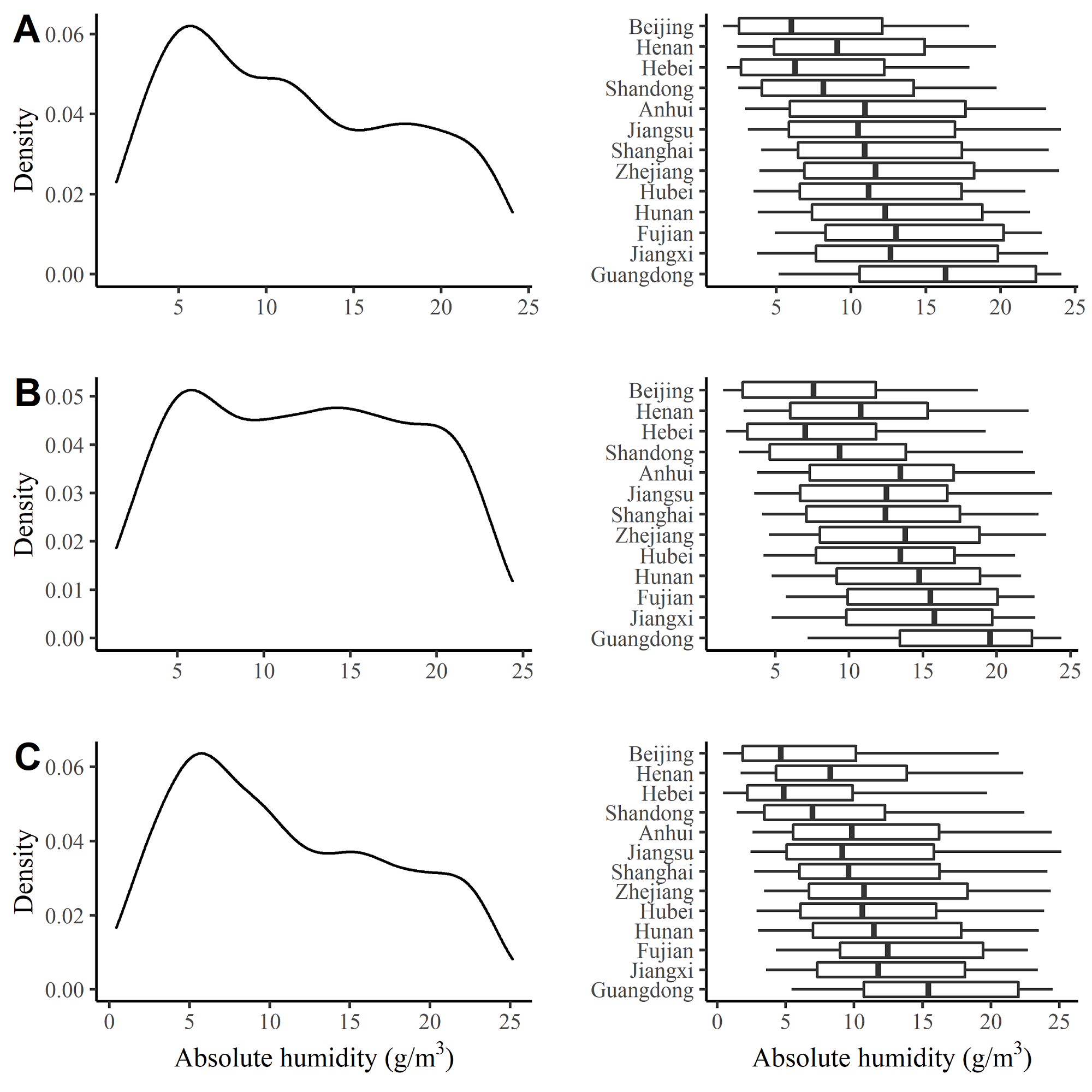

Supplement: S1 Fig — Overall distribution (left) and region-specific distribution (right) of absolute humidity during the epidemic period in the 2013/2014 (A), 2014/2015 (B) and 2016/2017 (C) influenza seasons. (TIF) [file ppat.1011046.s001.tif]

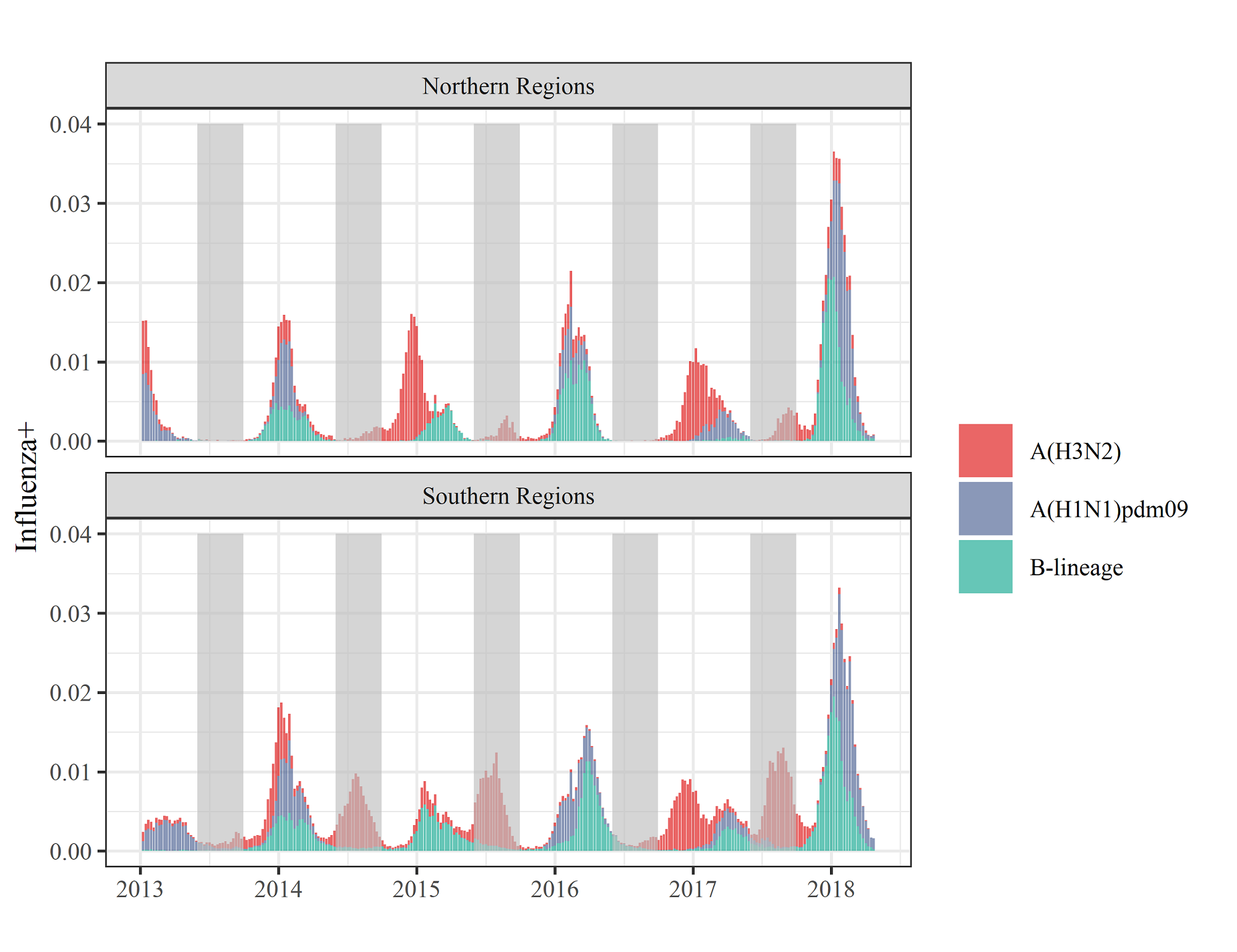

Supplement: S2 Fig — The influenza+ was calculated as the product of the influenza-like illness incidence rate, the proportion of influenza-like illness samples that tested positive for influenza and the subtype-specific proportion. Northern regions: Beijing, Hebei, Henan and Shandong. Southern regions: Guangdong, Jiangxi, Hunan, Hubei, Fujian, Zhejiang, Jiangsu, Anhui and Shanghai. The grey shadows represent the summer-autumn months (June, July, August and September) in China. (TIF) [file ppat.1011046.s002.tif]

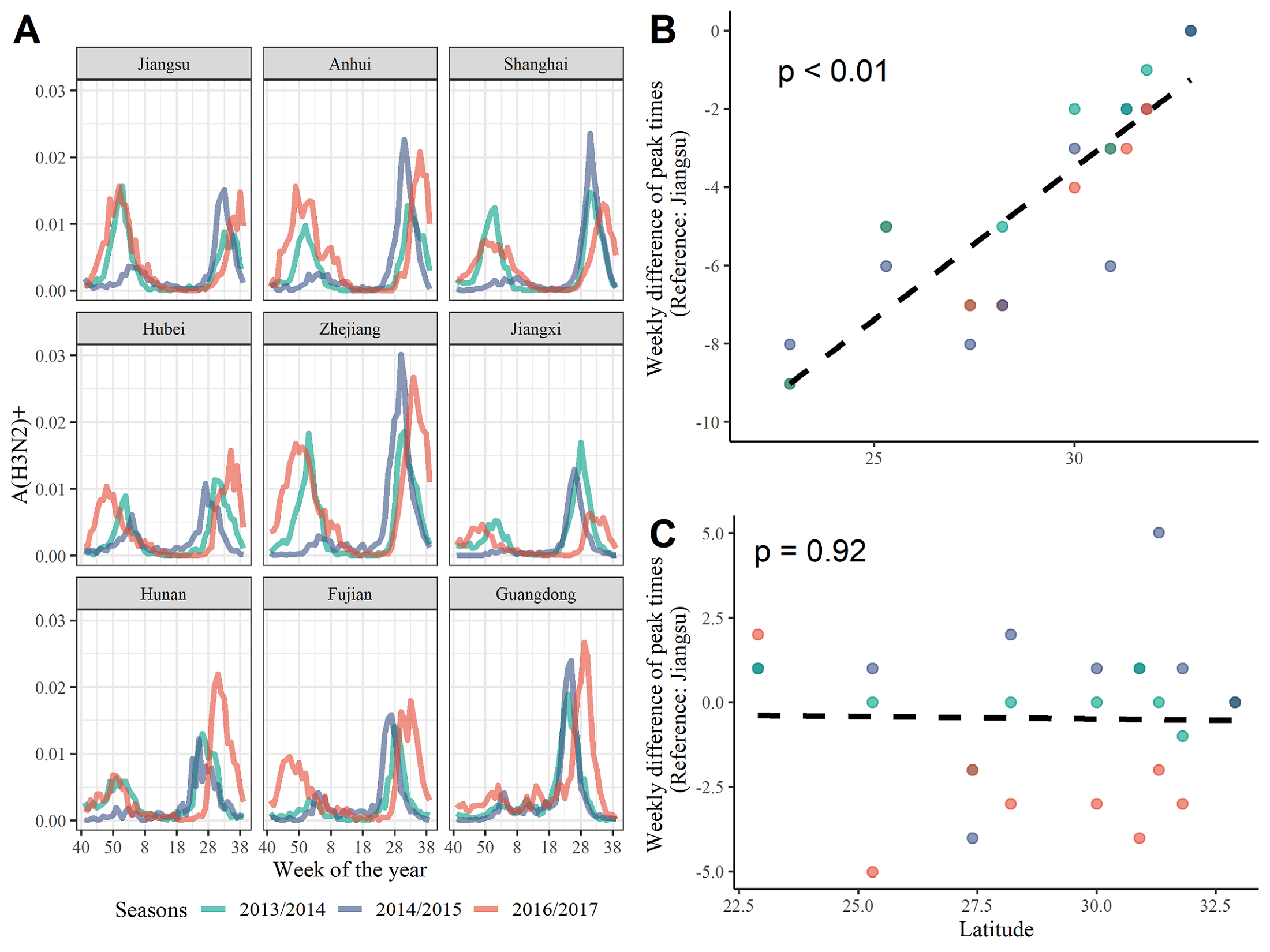

Supplement: S3 Fig — Seasons were color coded as green, blue and red for 2013/2014, 2014/2015 and 2016/2017 respectively. A) The time-series of A(H3N2)+ for nine selected regions from southern China. B) The relationship between the peak week of the summer epidemics and the latitude in nine southern regions of China. C) The relationship between the peak week of the winter epidemics and the latitude in nine southern regions of China. The most northern region Jiangsu among the selected nine regions was taken as the reference to calculate the weekly difference of peak times in B and C. A linear regression model was utilized here to explore the relationship between the weekly difference of peak times and latitude. The black dashed line in B and C represent the linear fitted line. A significant relationship between the weekly difference of peak times and latitude was observed in the summer epidemics (p < 0.01), but not in the winter epidemics (p = 0.92). (TIF) [file ppat.1011046.s003.tif]

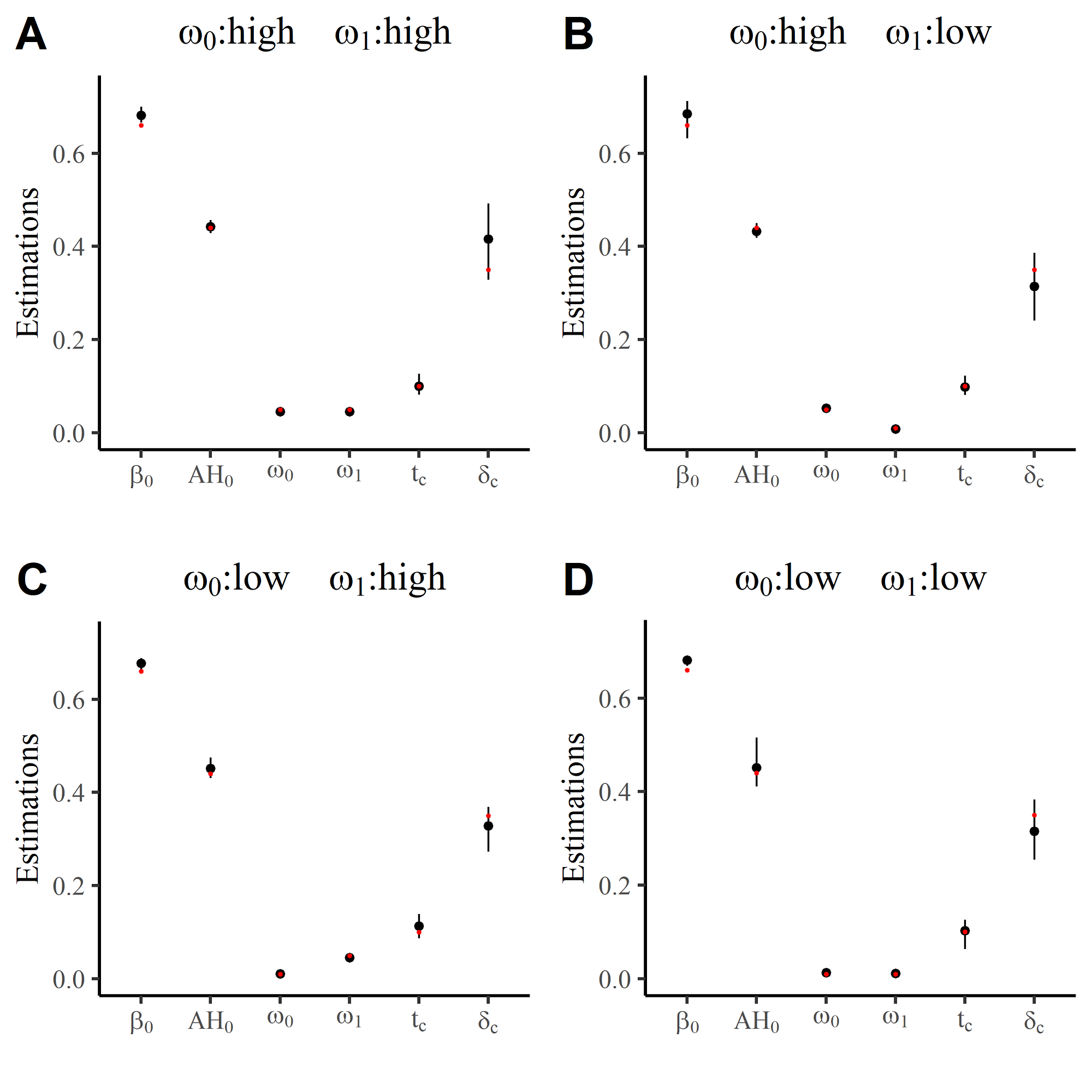

Supplement: S4 Fig — Four scenarios were tested here with the different relationship between absolute humidity (AH) and the transmission of influenza A(H3N2): the pre-defined values for ω0 and ω1 at either high (0.05) or low (0.01). If the value of ω0 and ω1 were set at the low and high values, respectively, the relationship between AH and A(H3N2) transmission was formulated as the “J” relationship. Other parameters and initial states were obtained from the maximum likelihood estimations in the meta-population transmission model for the influenza season 2013/2014. Based on the simulated time-series, the maximum likelihood estimation and its 95% confidence interval for the key parameters (black dots and lines in the figure) was re-estimated based on the inference framework and compared with the pre-defined value (red dots). (TIF) [file ppat.1011046.s004.tif]

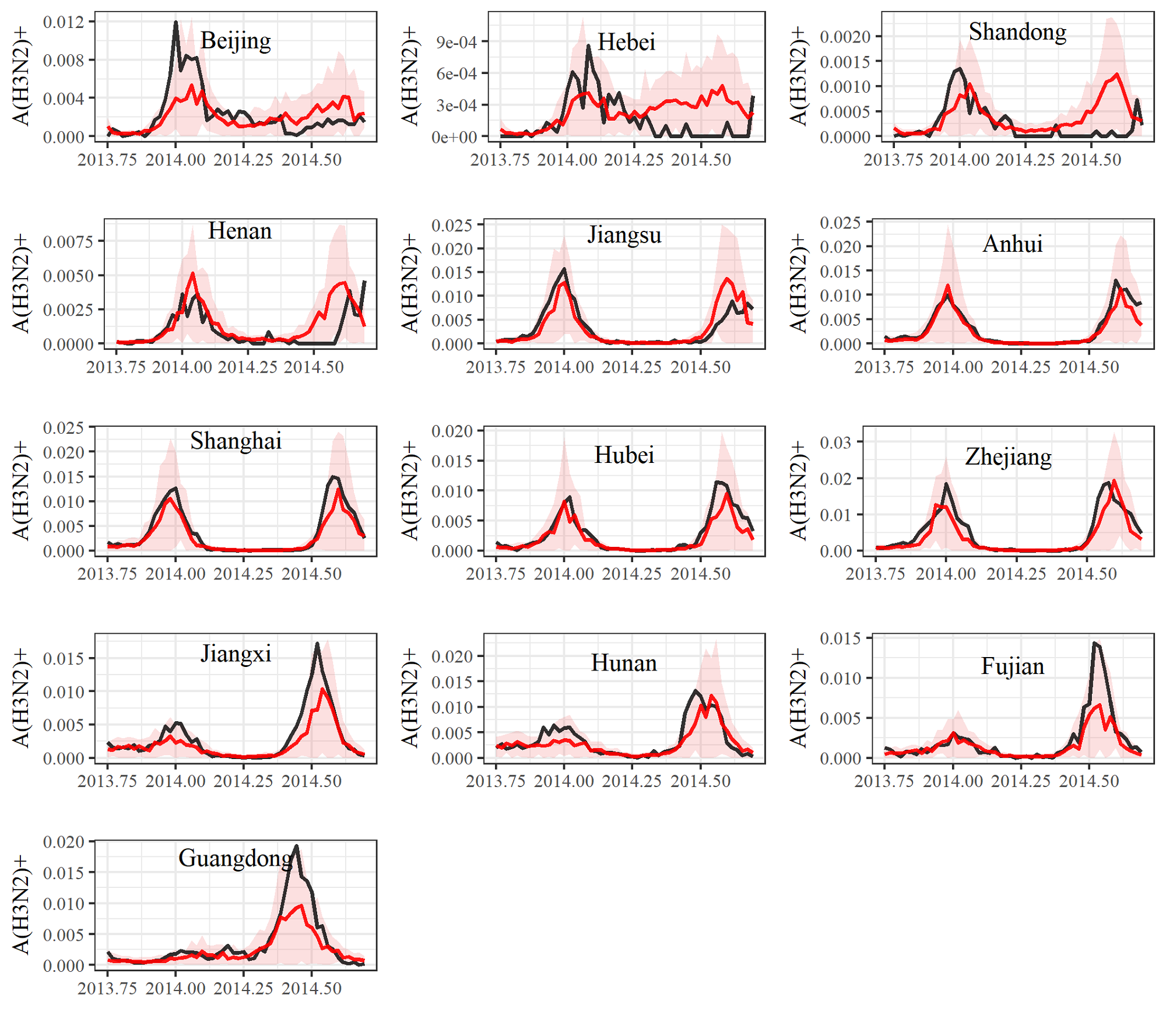

Supplement: S5 Fig — In total, thirteen regions were selected, including four regions (Beijing, Hebei, Shandong and Henan) without the obvious summer epidemics of influenza A(H3N2) virus. The black line represents the surveillance data, while the red one shows the median value of the simulated time-series based on the maximum likelihood estimation in the meta-population model with the 95% confidence interval (red shadows). (TIF) [file ppat.1011046.s005.tif]

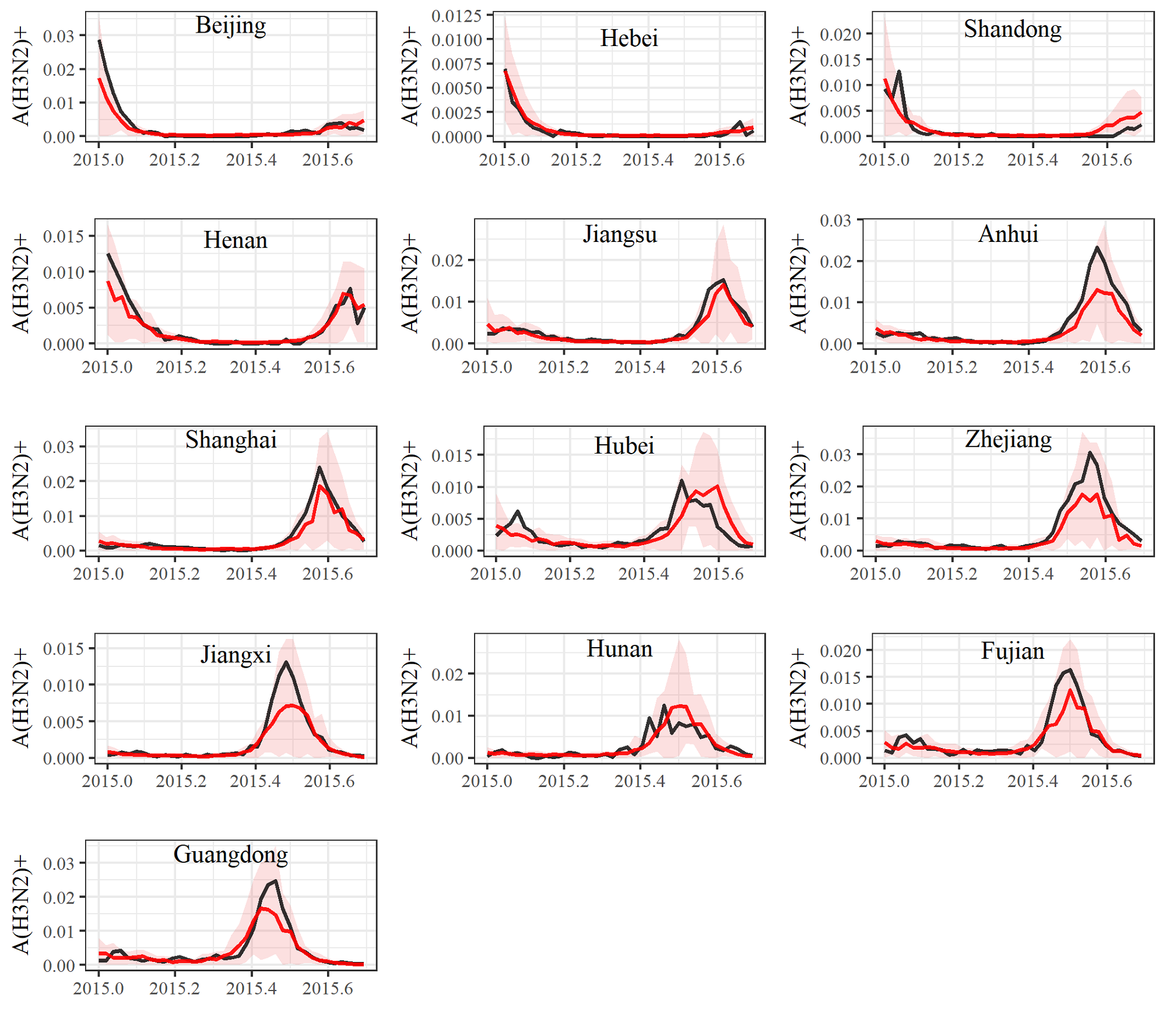

Supplement: S6 Fig — In total, thirteen regions were selected, including four regions (Beijing, Hebei, Shandong and Henan) without the obvious summer epidemics of influenza A(H3N2) virus. The black line represents the surveillance data, while the red one shows the median value of the simulated time-series based on the maximum likelihood estimation in the meta-population transmission model with the 95% confidence interval (red shadows). (TIF) [file ppat.1011046.s006.tif]

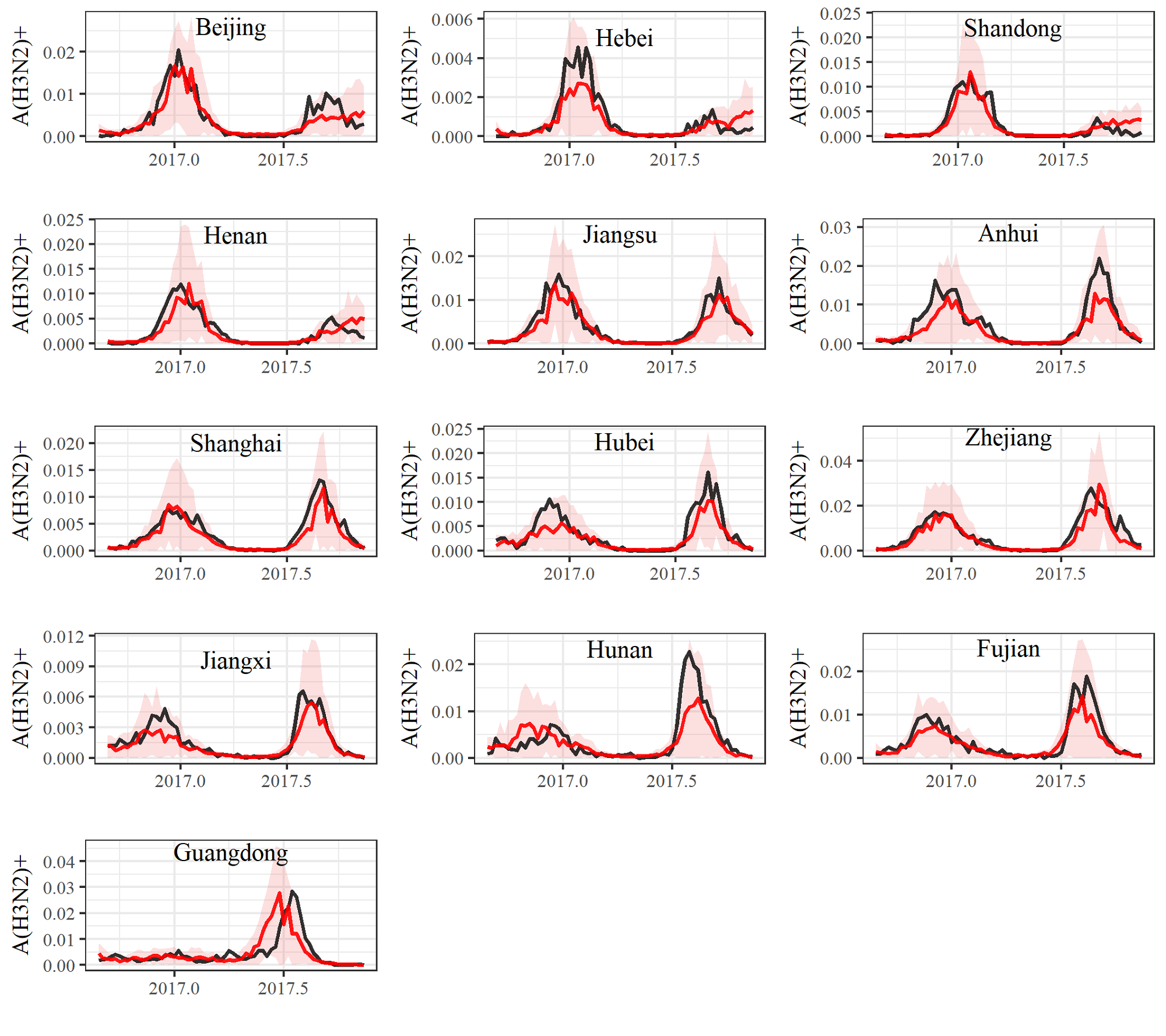

Supplement: S7 Fig — In total, thirteen regions were selected, including four regions (Beijing, Hebei, Shandong and Henan) without the obvious summer epidemics of influenza A(H3N2) virus. The black line represents the surveillance data, while the red one shows the median value of the simulated time-series based on the maximum likelihood estimation in the meta-population transmission model with the 95% confidence interval (red shadows). (TIF) [file ppat.1011046.s007.tif]

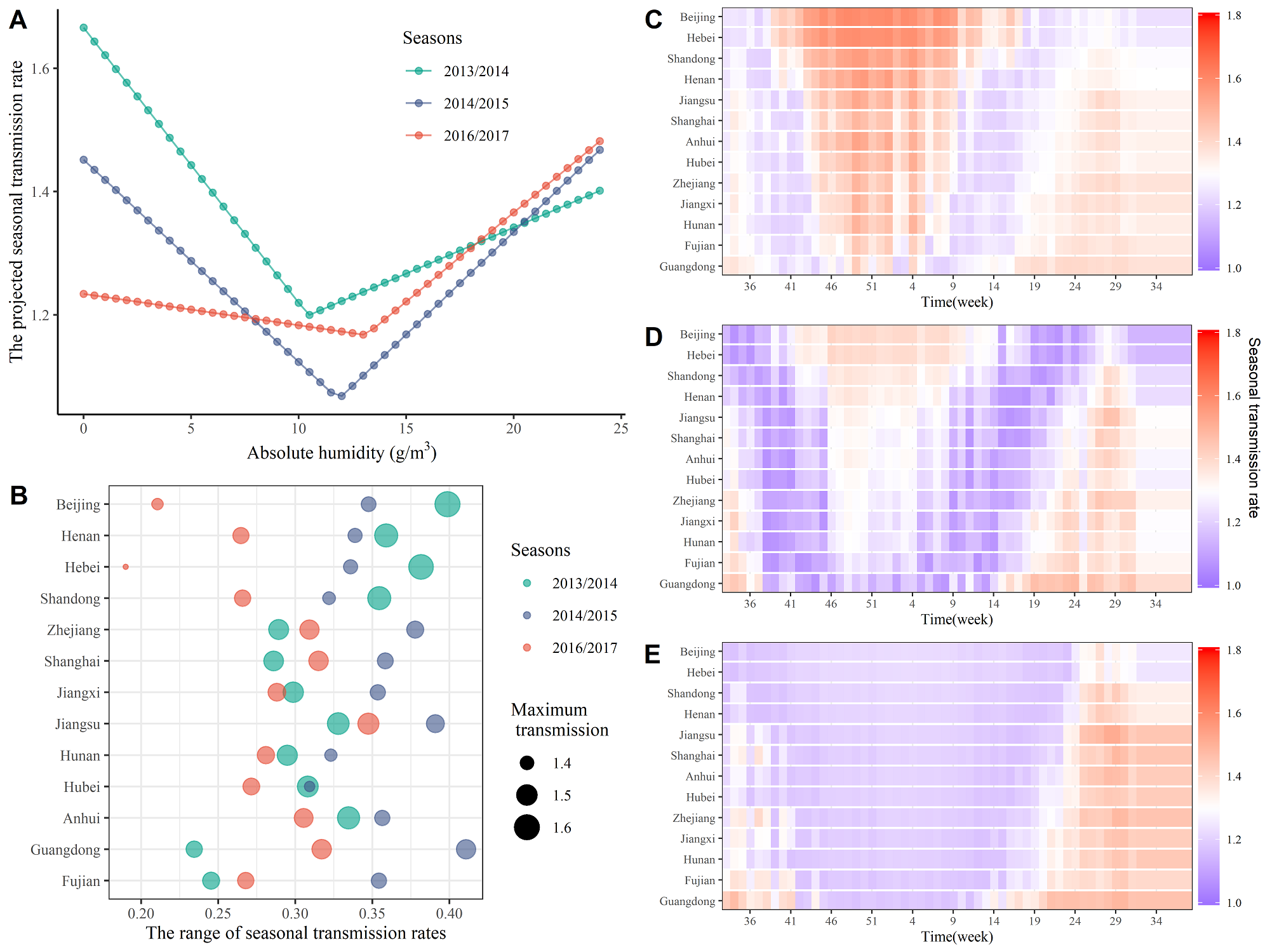

Supplement: S8 Fig — The seasonal transmission rate was nonlinearly formulated using the absolute humidity and four parameters (R0, ω0, ω1 and AH0) in the meta-population transmission model. Based on the maximum likelihood estimations, the seasonal transmission rates were projected. A) The nonlinear relationship between absolute humidity and the transmission of influenza A(H3N2). B) The geographical heterogeneity in the seasonal transmission rates of influenza A(H3N2). Seasons were color coded as green, blue and red for the 2013/2014, 2014/2015, and 2016/2017 influenza seasons, respectively. Based on the maximum likelihood estimations, the seasonal transmission rates were projected in the 2013/2014(C), 2014/2015(D), and 2016/2017(E) influenza seasons (see Material and Method). (TIF) [file ppat.1011046.s008.tif]

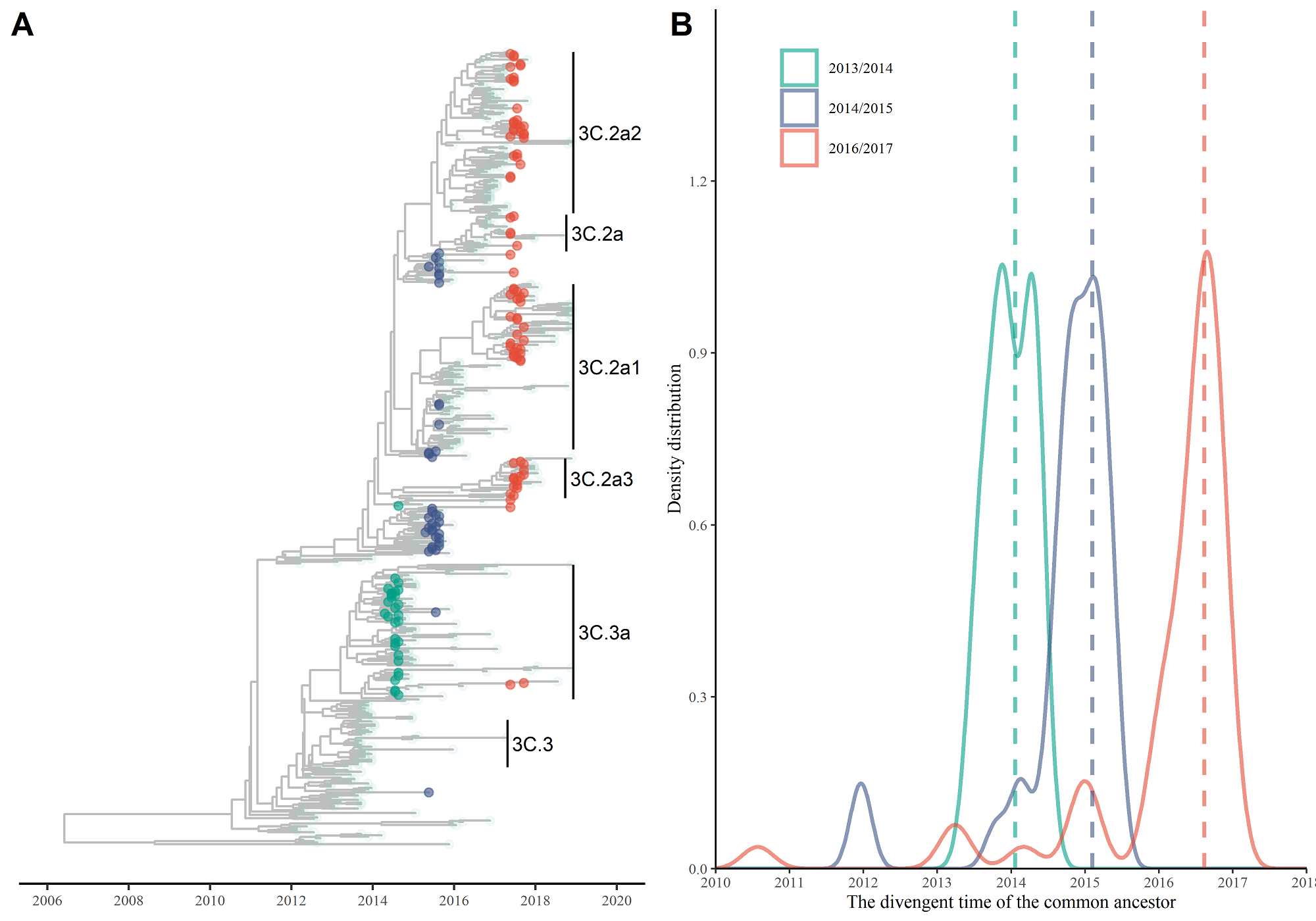

Supplement: S9 Fig — A) Bayesian time-scaled phylogenetic tree for influenza A(H3N2) virus in the selected regions of China between 2012 and 2018. Tip strains colored on the phylogenetic tree denote the strains in the summer-autumn months (June, July, August and September) in the 2013/2014 (green), 2014/2015 (brown), and 2016/2017 (red) influenza seasons, respectively. B) Distribution of the divergent times for the HA segment of influenza A(H3N2) virus. The solid lines represent the density distribution of the divergent times for HA of the influenza A(H3N2) virus, while the dashed lines show the estimated occurring times of the antigenic change from the meta-population transmission model (Table 1). (TIF) [file ppat.1011046.s009.tif]

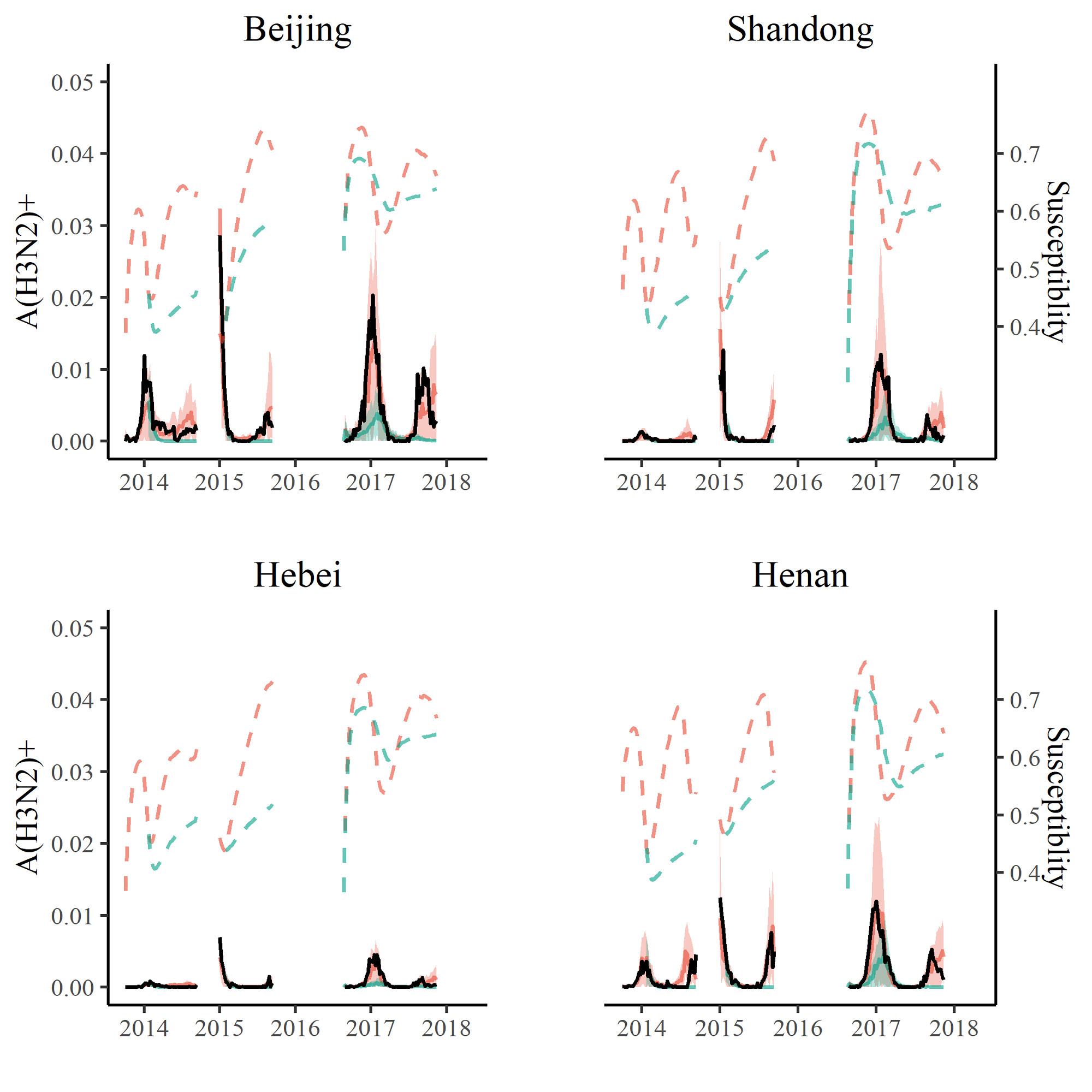

Supplement: S10 Fig — Based on the maximum likelihood estimations in the meta-population transmission model for the 2013/2014, 2014/2015, and 2016/2017 influenza seasons, the propagation of population susceptibility under two scenarios (with or without the antigenic antigenic) was obtained. The black line represents the surveillance data of A(H3N2)+. The red line and shadow represent the median value and its 95% confidence interval of A(H3N2)+ in the simulated time-series with the antigenic change, while the green one for the results without the antigenic change. The right y-axis represents the population susceptibility, which is calculated using the number of susceptible populations dividing the whole population in each region. (TIF) [file ppat.1011046.s010.tif]

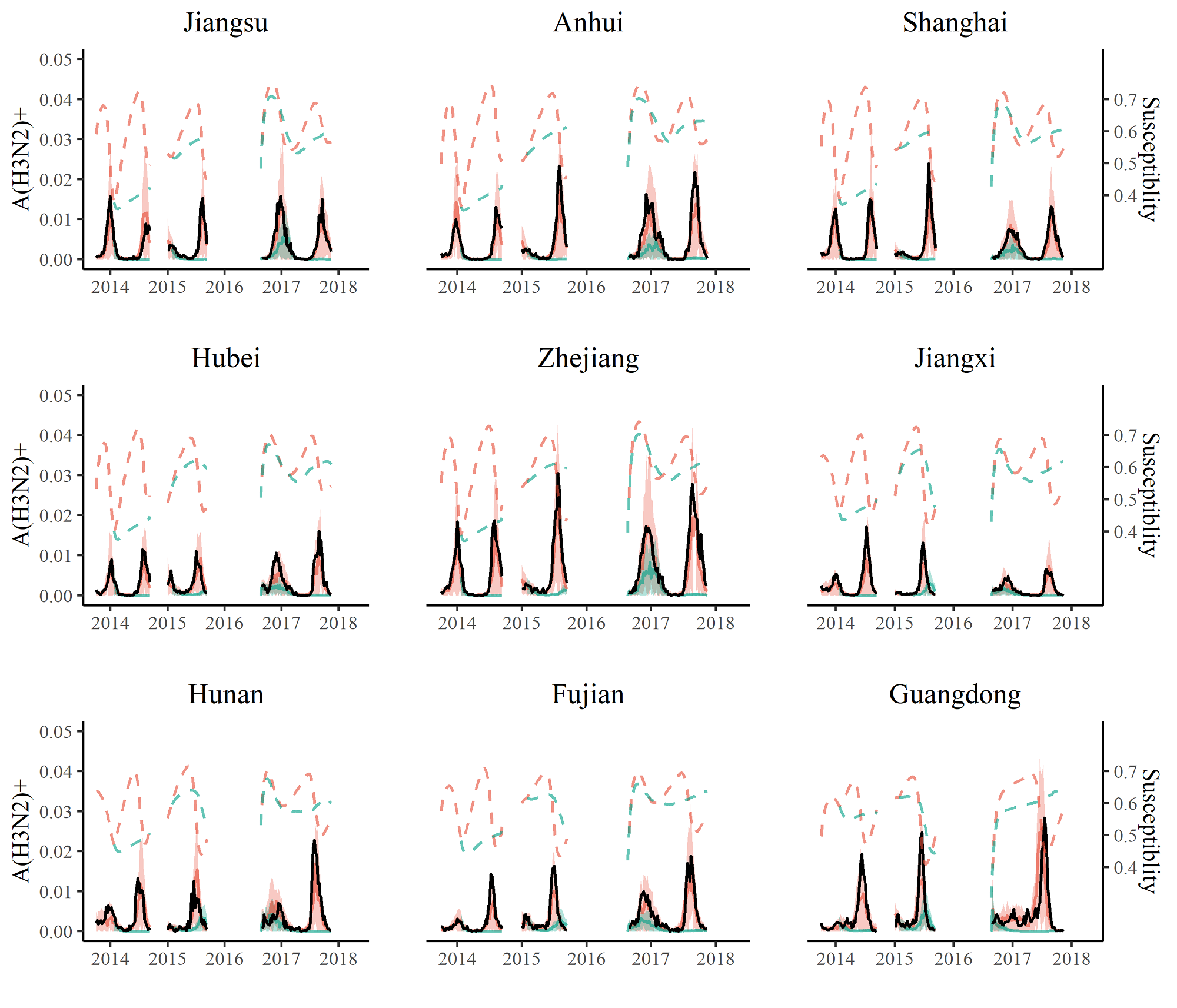

Supplement: S11 Fig — Based on the maximum likelihood estimations in the meta-population transmission model for the 2013/2014, 2014/2015, and 2016/2017 influenza seasons, the propagation of population susceptibility under two scenarios (with or without antigenic change) was obtained. The black line represents the surveillance data of A(H3N2)+. The red line and shadow represent the median value and its 95% confidence interval of A(H3N2)+ in the simulated time-series with the antigenic change, while the green one is for the results without the antigenic change. The right y-axis represents the population susceptibility, which is calculated using the number of susceptible populations dividing the whole population in each region. (TIF) [file ppat.1011046.s011.tif]

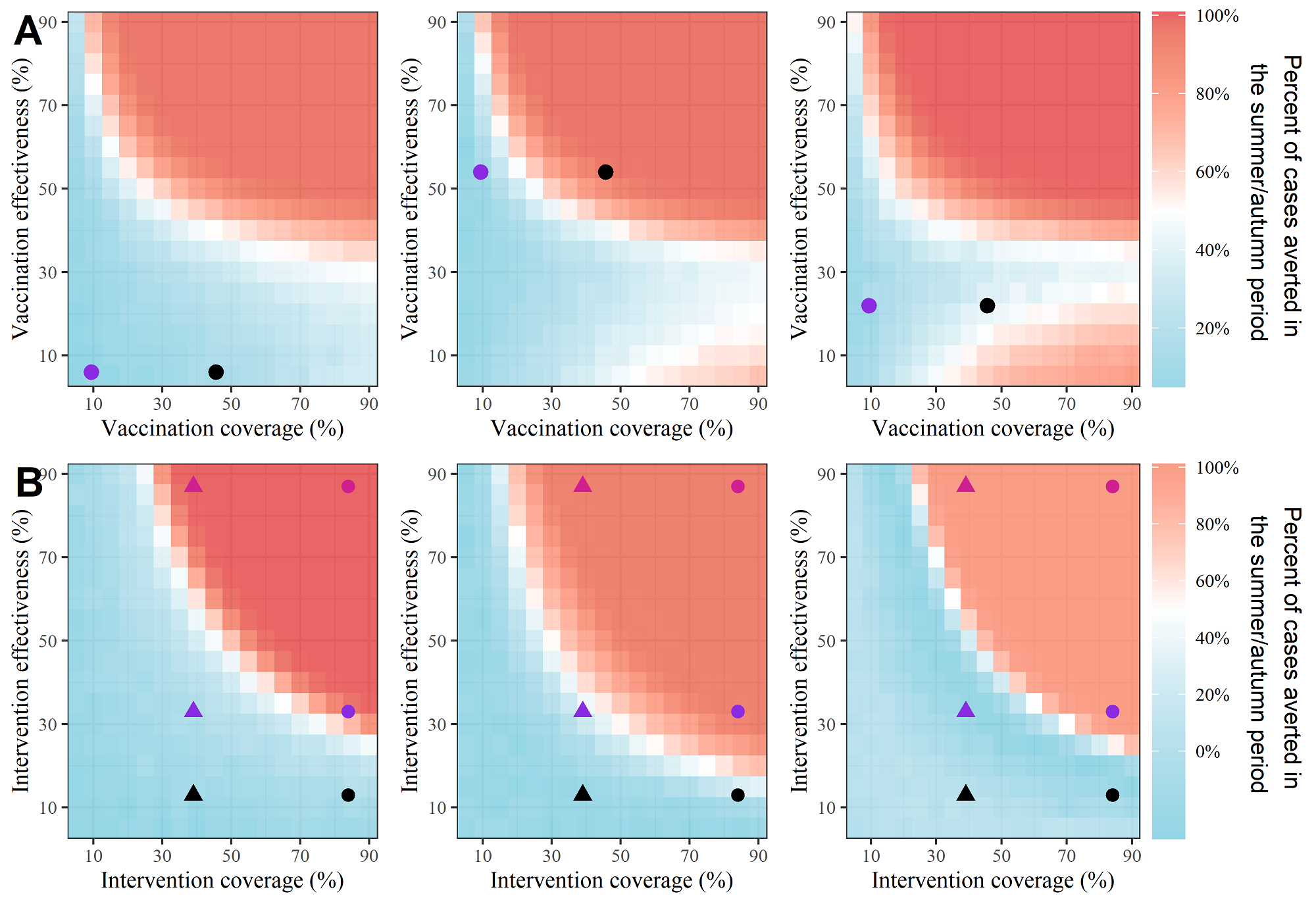

Supplement: S12 Fig — The coverage and effectiveness of interventions range from 5% to 90% with an interval of 5%. The triangles and circles in the top panel (A) represent the influenza vaccine coverage in China (9.4% [9]) and United States (43.8% [10]), respectively. Different colors represent the average (black, 29%) and season-specified vaccine effectiveness for influenza A(H3N2) (blue,0.09 for 2014/2015 [11], 0.54 for 2015/2016 [12] and 0.22 for 2017/2018 [13]. The vaccine effectiveness in the summer epidemic was assumed based on the estimated vaccine effectiveness in the following season). The triangles and circles in the bottom panel (B) show the percentage of the population using facemasks plus hand hygiene in the non-pandemic period (39% [14]) and pandemic period (84% [15]), respectively, while the color in the triangles or circles represents the low (13%, black), middle (23%, blue) and high (87.0%, red) effectiveness of using facemasks plus hand hygiene to prevent influenza A(H3N2) [16], respectively. (TIF) [file ppat.1011046.s012.tif]

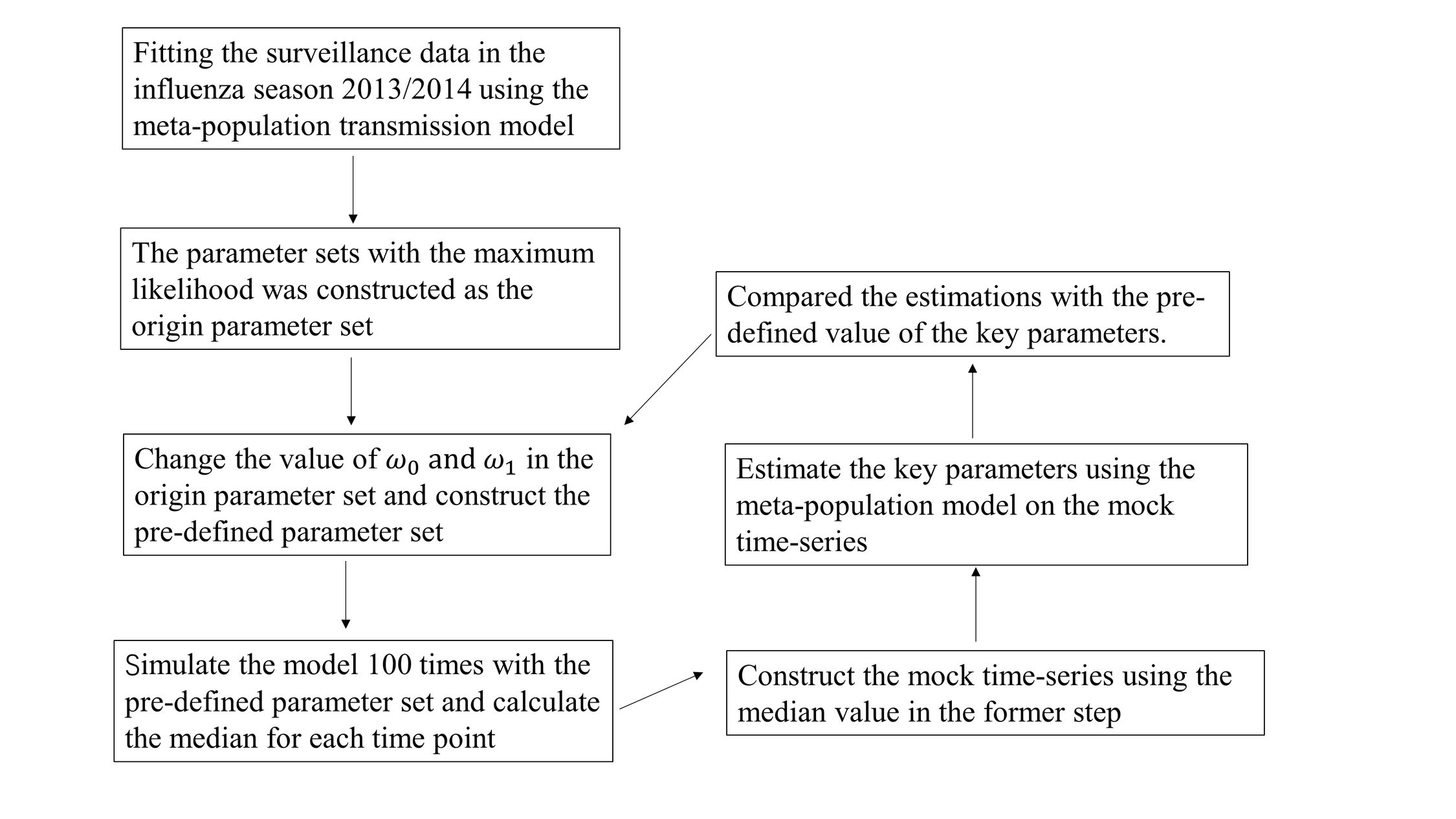

Supplement: S13 Fig — (TIF) [file ppat.1011046.s013.tif]
