# Supplementary material for: Mechanisms for the circulation of influenza A(H3N2) in China: A spatiotemporal modelling study
Source: PLoS Pathog. 2022 Dec 16;18(12):e1011046. doi: 10.1371/journal.ppat.1011046 (PMC9803318; doi:10.1371/journal.ppat.1011046)
Supplement: S1 Table — (DOCX) [file ppat.1011046.s014.docx]

**S1 Table. Description of parameters in the meta-population transmission model**

| **Parameter** | **Definition** | **Value** |
| --- | --- | --- |
| $\mu$ | Death rate | 1/76 |
| $B_{i}$ | The birth population for region $i$ | Fixed |
| $N_{i}$ | The total population for region $i$ | Fixed |
| $\rho_{0}^{'}$ | The basic reporting probability for other influenza subtypes infections  (A(H1N1)pdm09 and B-lineage) | Estimated |
| $\upsilon_{i}$ | The regional scale factor for the reporting probability | Estimated |
| $\xi^{'}$ | the rate of immunity waning for the population infected by other influenza subtypes (A(H1N1)pdm09 and B-lineage) | Estimated |
| G | The gravitation constant in the gravity model | Estimated |
| $\omega_{0}$ | The coefficient of absolute humidity (AH) on A(H3N2) transmission when AH is smaller than ${AH}_{0}$ | Estimated |
| $\omega_{1}$ | The coefficient of absolute humidity (AH) on A(H3N2) transmission when AH is larger than ${AH}_{0}$ | Estimated |
| ${AH}_{0}$ | The threshold value for AH where the lowest seasonal transmission rate is observed | Estimated |
| $\beta_{0}$ | The lowest seasonal transmission rate of A(H3N2) virus | Estimated |
| $\epsilon$ | The amplitude of the holiday effect | Estimated |
| $t_{c}$ | The occurring time when the properties of A(H3N2) virus changed | Estimated |
| $\delta_{ca}$ | The effect of changes in viral properties on transmissibility | Estimated |
| $\delta_{ct}$ | The effect of changes in viral properties on immunity waning | Estimated |
| $\rho_{0}$ | The basic reporting probability of A(H3N2) infections | Estimated |
| $\varrho$ | The reporting over-dispersion of A(H3N2) infections | Estimated |
| $S_{0,i}$ | The initial susceptible population for region $i$ | Estimated |
| $I_{0,i}$ | The initial infected population for region $i$ | Estimated |
| $R_{0,i}$ | The initial recovery population from the A(H3N2) infections for region $i$ | Estimated |
| $R_{0,i}^{‘}$ | The initial recovery population infected by A(H1N1)pdm09 and B-lineage for region $i$ | Estimated |
| $1/\gamma$ | The infectious period | 3 days |
| $1/\xi_{0}$ | The recovery period for the population infected by influenza A(H3N2) | 6 years |
