# Supplementary material for: Mechanisms for the circulation of influenza A(H3N2) in China: A spatiotemporal modelling study
Source: PLoS Pathog. 2022 Dec 16;18(12):e1011046. doi: 10.1371/journal.ppat.1011046 (PMC9803318; doi:10.1371/journal.ppat.1011046)
Supplement: S2 Table — (DOCX) [file ppat.1011046.s015.docx]

**S2 Table. Model tests for the effect of viral mutations on the dynamics of**

**influenza A(H3N2) (Akaike information criterion)**

| Influenza seasons | No  Change | One change in the immunity waning | One change in the  transmission | Two changes in the immunity waning |
| --- | --- | --- | --- | --- |
| 2013/2014 | 18464.68 | 14684.28 | 14864.67 | 14691.85 |
| 2014/2015 | 11417.03 | 11411.47 | 11456.47 | ----- |
| 2016/2017 | 19921.99 | 19658.03 | 19703.74 | 19673.60 |

Note: The meta-population transmission models are constructed for the 2013/2014, 2014/2015 and 2016/2017 influenza seasons. Four scenarios are tested for the 2013/2014 and 2016/2017 influenza seasons, where the winter epidemic and summer epidemic coexist. Since there is only the summer epidemic in the 2014/2015 influenza season, therefore, the two breakpoints scenario is unlikely to occur. As a result, only three scenarios are tested. A change in the immunity waning means the rate of immunity waning is increased due to antigenic change, and one change in the transmission means the time-varying transmission rate is increased due to the viral mutations. Two changes in the immunity waning mean the rate of immunity waning is increased both in the winter and summer epidemics (**see Material and Methods**).
