# Supplementary material for: Mechanisms for the circulation of influenza A(H3N2) in China: A spatiotemporal modelling study
Source: PLoS Pathog. 2022 Dec 16;18(12):e1011046. doi: 10.1371/journal.ppat.1011046 (PMC9803318; doi:10.1371/journal.ppat.1011046)
Supplement: S3 Table — (DOCX) [file ppat.1011046.s016.docx]

**S3 Table.** **Model fitting for the meta-population model under different scenarios**

**(Maximum likelihood value)**

| Influenza seasons | Basic model | Basic model without antigenic change | Basic model without environmental drivers | Basic model without holiday effect |
| --- | --- | --- | --- | --- |
| 2013/2014 | -7252 | -9416 | -7333 | -7260 |
| 2014/2015 | -5599 | -5627 | -5712 | -5606 |
| 2016/2017 | -9740 | -11033 | -10072 | -9818 |

Note: The basic meta-population transmission model is the model incorporates the effects of antigenic change, environmental drivers and holiday term. The basic model without the effect of antigenic change defines the value of the parameter $\delta_{ct}$as 1; The basic model without the effects of environmental drivers defines the values of the parameters $\omega_{0}$ and $\omega_{1}$ as 0; The basic model without the effect of the holiday term defines the value of the parameter $\epsilon$ as 0.
