# Supplementary material for: Mechanisms for the circulation of influenza A(H3N2) in China: A spatiotemporal modelling study
Source: PLoS Pathog. 2022 Dec 16;18(12):e1011046. doi: 10.1371/journal.ppat.1011046 (PMC9803318; doi:10.1371/journal.ppat.1011046)
Supplement: S1 Text — (DOCX) [file ppat.1011046.s017.docx]

**S1 Text**

**Model validation for the meta-population transmission model**

Before the meta-population transmission model was used to fit the surveillance data, the inference framework was validated using the mock time series simulated from the meta-population transmission model with the pre-defined parameters. To examine whether the meta-population transmission model could uncover the complex climatic dependence of influenza A(H3N2) virus, four scenarios were tested here with the different relationship between absolute humidity (AH) and the transmission of influenza A(H3N2). Since the parameters $\omega_{0}$ and $\omega_{1}$ respectively decides the strength of A(H3N2) transmission in the low-humidity and high-humidity situation, thus, four different kinds of relationship (V-shaped, J-shaped, inverted J-shaped and weak one) between AH and the transmission of influenza A(H3N2) can be formulated with the values for $\omega_{0}$ and $\omega_{1}$ at either high (0.05) or low (0.01). For instance, if the values of $\omega_{0}$ and $\omega_{1}$ were set at the low and high one, respectively, the relationship between AH and the transmission of influenza A(H3N2) was formulated as the J-shaped one. Other model parameters were obtained from the maximum likelihood estimations from the meta-population model on the 2013/2014 influenza season. The focused parameters ($\beta_{0}$, ${AH}_{0}$, $\omega_{0}$,$\omega_{1}$, $\delta_{ca}$ and$t_{c}$, **S1** **Table**) were re-estimated using the inference framework on the mock time-series. The efficacy of the inference framework was evaluated by checking whether the estimated confidence interval contains the originally defined parameter values. The pipeline for the model validation was in **S13 Fig**.

**Phylogenetic analysis**

To quantify the evolution pattern of influenza A(H3N2) in China, a phylogenetic analysis of the HA segment between 2012 and 2018 was performed. For each segment, alignments were created with MUSCLE v3.7 using default settings and the undetermined nucleotides were replaced by gaps [1]. The sequences with no detailed location and no collection date information were deleted. For the paired sequence with the hamming distance equal to zero in the same influenza season, only one sequence was retained in the further analysis. For each region, sequences were randomly down-sampled to three each month to control the sampling biases and alleviate the computational requirement, resulting in 537 sequences.Based on the nucleotide sequence alignments, the initial maximum likelihood tree was inferred using the R treedater package [2], and strains overly divergent based on the root-to-tips distances were manually removed. Finally, the time-resolved phylogenetic tree was inferred using the Bayesian methods available in BEAST version 1.10.1 [3] and incorporated the SRD06 nucleotide substitution model and a strict molecular clock across branches [4]. Tracer v1.7.1 was used to evaluate the stationarity of the Markov Chain Monte Carlo trace files and TreeAnnotator v1.10.4 was used to produce the maximum clade credibility trees [4, 5]. All the phylogenetic tree visualizations were constructed using the R package ggtree [6].

**Contribution of driving factors on the dynamics of influenza A(H3N2)**

To partition the variation in the dynamics of influenza A(H3N2) into portions attributable

to the initial population susceptibility (population susceptibility at the onset time), climatic factors, and antigenic change, a simulation experiment with the factorial design was utilized [7]. Based on the maximum likelihood estimations for these three influenza seasons, the time-series under two different scenarios (informed by the driving factor or not) were simulated.

And then, the weekly surveillance data for each region was fitted using the median values from the simulation in a generalized linear model and the coefficient of determination ($R^{2}$) was calculated, which used the variance function to measure the proportion of variation in the dependent variable explained by the predictors. That is, the variation of the surveillance data explained by the simulated time-series informed or not informed by the driving factor denotes as $R_{y}^{2}$ and $R_{n}^{2}$, respectively. Finally, the variation in the dynamics of influenza A(H3N2) attributable to the driving factor ($\Delta R^{2}$) was calculated as follows:

$$\Delta R^{2}= R_{y}^{2}- R_{n}^{2}$$

To gain the simulated time-series informed by the driver (initial population susceptibility, climatic factor, and antigenic change), driver-related parameters were maintained at the estimated level. To obtain the simulated time-series that was not informed by the initial population susceptibility or climatic factor, the initial condition (the estimated percentage of S, I, R and $R^{'}$ at the onset time) or absolute humidity was randomly shuffled among regions before being taken into the meta-population transmission model. To gain the simulated time-series that was not informed by antigenic change, the rate of immunity waning ($\xi$) was maintained at the basic level through the study period (1/6 years^-1^, **S1 Table**).

**Effects of interventions on the summer epidemics of influenza A(H3N2)**

To examine the effects of the non-pharmaceutical intervention (using facemasks plus hand hygiene) and the pharmaceutical intervention (vaccination program) on the summer epidemics of influenza A(H3N2), counterfactual simulations with the specific setting on either the model compartment or the transmission process were constructed. Specifically, to explore the effect of vaccination campaign on the dynamics of influenza A(H3N2), a susceptible-vaccinated-infected-recovery-susceptible (SVIRS) model was utilized, which divided the whole population into the susceptible (S), the vaccinated (V), the infected (I), the recovery from influenza A(H3N2) infection (R), and the recovery population infected other influenza subtypes ($R^{'}$). A leakiness mode was utilized here regarding the aspects of vaccine protection, that is, the vaccine reduces, but does not eliminate, the potential for A(H3N2) infections [8]. The vaccine effectiveness was measured as the reduced probability of infection for the vaccinated relative to the unvaccinated when the level of virus exposure is the same [8]. The detailed differential equations were formulated as follows:

$$\frac{dS_{i}}{dt}=B_{i}- \lambda_{i}\left( t \right)S_{i}+\xi R_{i}- \frac{\Lambda_{i,t}}{\rho_{i}^{'}}+R_{i}^{'}\xi^{'}-\nu S_{i}-\mu S_{i}$$

$$\frac{dV_{i}}{dt}= \nu S_{i}+ \nu R_{i}+{\nu R}_{i}^{'}- (1-\varsigma)\lambda_{i}\left( t \right)S_{i}-\mu V_{i}$$

$$\frac{dI_{i}}{dt}= \left( 1-\varsigma\right)\lambda_{i}\left( t \right)S_{i}+ \lambda_{i}\left( t \right)S_{i}- \gamma I-\mu I_{i}$$

$\frac{dR_{i}}{dt}= \gamma I_{i}-\xi R_{i}-\mu R_{i}-\nu R_{i} (3)$

$$\frac{dR_{i}^{'}}{dt}= \frac{\Lambda_{i}}{\rho_{i}^{'}}-R_{i}^{'}\xi^{'}i-\mu R_{i}^{'}-{\nu R}_{i}^{'}$$

$$N_{i}=S_{i}+I_{i}+R_{i}+ R_{i}^{'}+V_{i}$$

Where $\nu$ and $\varsigma$ represent the vaccination coverage and vaccine effectiveness, respectively. Other parameters can be found in **S1 Table**. The vaccination campaign was assumed to be implemented on the last day of the first year in each influenza season (e.g., 31 December 2013 in the 2013/2014 influenza season). The vaccination coverage and vaccine effectiveness range from 5% to 90% with an interval of 5%. The vaccination coverage and vaccine effectiveness combined with other parameters estimated from the meta-population transmission model, formed the original parameters.

As for the non-pharmaceutical intervention (using facemasks plus hand hygiene), the original model structure (**see Materials and Methods: Meta-population mechanistic transmission model**) was utilized but with a specific setting on the transmission process. Here, the intervention was assumed to be implemented throughout the study period, and the time-varying transmission rate was formulated in the following manner: $\beta_{i}(t)$*(1- intervention coverage*intervention effectiveness). The intervention coverage and intervention effectiveness range from 5% to 90% with an interval of 5%. The intervention coverage and intervention effectiveness combined with other parameters estimated from the meta-population transmission model, formed the original parameters.

Based on the original parameters and meta-population transmission model, the simulated time-series were obtained. Here, the effects of interventions were tested only on the summer epidemics of influenza A(H3N2). A summer epidemic is defined as one in which the peak occurs in the summer-autumn months (June, July, August and September) and the peak intensity of A(H3N2)+ is greater than 0.005.

**References**

1. Edgar RC. MUSCLE: multiple sequence alignment with high accuracy and high throughput. Nucleic Acids Res. 2004;32(5):1792-7.

2. Volz EM, Frost SDW. Scalable relaxed clock phylogenetic dating. Virus Evolution. 2017;3(2).

3. Suchard MA, Lemey P, Baele G, Ayres DL, Drummond AJ, Rambaut A. Bayesian phylogenetic and phylodynamic data integration using BEAST 1.10. Virus Evol. 2018;4(1):vey016.

4. Bedford T, Riley S, Barr IG, Broor S, Chadha M, Cox NJ, et al. Global circulation patterns of seasonal influenza viruses vary with antigenic drift. Nature. 2015;523(7559):217-20.

5. Rambaut A, Drummond AJ, Xie D, Baele G, Suchard MA. Posterior summarization in Bayesian phylogenetics using Tracer 1.7. Systematic Biology. 2018;67(5):901-4.

6. Yu G, Smith DK, Zhu H, Guan Y, Lam TT-Y. ggtree: an r package for visualization and annotation of phylogenetic trees with their covariates and other associated data. Methods in Ecology and Evolution. 2017;8(1):28-36.

7. Oidtman RJ, Lai S, Huang Z, Yang J, Siraj AS, Reiner RC, Jr., et al. Inter-annual variation in seasonal dengue epidemics driven by multiple interacting factors in Guangzhou, China. Nat Commun. 2019;10(1):1148.

8. Magpantay FMG, Domenech De Cellès M, Rohani P, King AA. Pertussis immunity and epidemiology: mode and duration of vaccine-induced immunity. Parasitology. 2016;143(7):835-49.

9. Wang Q, Yue N, Zheng M, Wang D, Duan C, Yu X, et al. Influenza vaccination coverage of population and the factors influencing influenza vaccination in mainland China: A meta-analysis. Vaccine. 2018;36(48):7262-9.

10. Centers for Disease Control and Prevention. Flu vaccination coverage, United States. Available: <https://www.cdc.gov/flu/fluvaxview/coverage-by-season.htm>.

11. Zimmerman RK, Nowalk MP, Chung J, Jackson ML, Jackson LA, Petrie JG, et al. 2014-2015 influenza vaccine effectiveness in the United States by vaccine type. Clin Infect Dis. 2016;63(12):1564-73.

12. Zhang L, Pan Y, Hackert V, van der Hoek W, Meijer A, Krafft T, et al. The 2015-2016 influenza epidemic in Beijing, China: Unlike elsewhere, circulation of influenza A(H3N2) with moderate vaccine effectiveness. Vaccine. 2018;36(33):4993-5001.

13. Rolfes MA, Flannery B, Chung JR, O'Halloran A, Garg S, Belongia EA, et al. Effects of influenza vaccination in the United States during the 2017-2018 influenza season. Clin Infect Dis. 2019;69(11):1845-53.

14. Chan EY, Cheng CK, Tam G, Huang Z, Lee P. Knowledge, attitudes, and practices of Hong Kong population towards human A/H7N9 influenza pandemic preparedness, China, 2014. BMC Public Health. 2015;15:943.

15. Lau J, Yu Y, Xin M, She R, Luo S, Li L, et al. Adoption of preventive measures during the very early phase of the COVID-19 outbreak in China: national cross-sectional survey study. JMIR Public Health Surveill. 2021;7(10):e26840.

16. Cowling BJ, Chan Kh Fau - Fang VJ, Fang Vj Fau - Cheng CKY, Cheng Ck Fau - Fung ROP, Fung Ro Fau - Wai W, Wai W Fau - Sin J, et al. Facemasks and hand hygiene to prevent influenza transmission in households: a cluster randomized trial. Ann Intern Med. 2009;151(7):437-46.
